# Electrolyte–Framework Matching in High‐Voltage TEMPO‐COF Cathodes for Lithium Batteries

**DOI:** 10.1002/advs.76112

**Published:** 2026-06-18

**Authors:** Marilyn Esclance DMello, Nagaraj Patil, Fanni Fekecs, Sergio Pinilla, Rebeca Marcilla, Manuel Souto

**Affiliations:** ^1^ CiQUS Centro Singular de Investigación en Química Biolóxica e Materiais Moleculares Departamento De Química‐Física Universidade De Santiago de Compostela Santiago de Compostela Spain; ^2^ Electrochemical Processes Unit IMDEA Energy Móstoles Spain; ^3^ Oportunius, Galician Innovation Agency (GAIN) Santiago de Compostela Spain

**Keywords:** Li‐organic batteries, organic batteries, organic cathodes, redox‐active covalent organic frameworks, TEMPO radical

## Abstract

High‐voltage organic cathodes based on stable nitroxyl radicals are promising candidates for sustainable energy storage. However, porous TEMPO‐based frameworks remain underdeveloped compared to linear polymers, and the role of electrolyte anions in governing their performance is poorly understood. Herein, two imine‐linked covalent organic frameworks (COFs) are post‐synthetically functionalized with N_3_‐TEMPO via click chemistry, affording crystalline, porous **TEMPO‐TB** and **TEMPO‐TP** COFs with uniformly distributed redox‐active sites. When evaluated as cathodes for Li‐organic batteries, both materials exhibit reversible p‐type redox activity at ∼3.6–3.7 V vs. Li/Li^+^. A systematic comparison of LiX electrolytes (X = PF_6_^−^, ClO_4_^−^, BF_4_^−^, DFOB^−^, and TFSI^−^) in carbonate‐based media reveals strong anion‐dependent electrochemical behavior. Among the electrolytes studied, LiDFOB provides the best balance of capacity, rate capability, and cycling stability, attributed to favorable anion‐coupled charge storage, interfacial charge‐transfer behavior, and pseudocapacitive contributions. Binder‐free buckypaper electrodes enable up to 80 wt.% active material and mass loadings of 40 mg cm^−2^, while maintaining ∼1.3 mAh cm^−2^. This represents the highest reported mass loading and areal capacity for TEMPO‐based cathodes in lithium batteries. These results establish framework–electrolyte matching as a key design principle for high‐voltage TEMPO‐based cathodes and provide new guidance for the development of radical‐functionalized organic battery materials.

## Graphical abstract

TEMPO‐functionalized covalent organic frameworks are investigated as high‐voltage cathodes for lithium batteries. Screening five electrolyte anions identifies lithium difluoro(oxalato)borate (LiDFOB) as the optimal electrolyte, balancing capacity, rate capability, and cycling stability, while buckypaper electrodes achieve high mass loading (40 mg cm^−2^) and an areal capacity of 1.3 mAh cm^−2^.

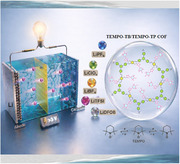

## 1 Introduction

The development of sustainable high‐voltage organic cathodes remains a central challenge in rechargeable battery research, because it is still difficult to combine high redox potential, structural robustness, fast ion transport, and long‐term cycling stability within a single organic platform [1, 2, 3, 4]. In this context, covalent organic frameworks (COFs), a class of crystalline porous organic polymers constructed from organic building blocks linked through robust covalent bonds, have emerged as attractive electrode materials for energy storage [5, 6, 7, 8]. Their long‐range order, permanent porosity, structural modularity, and insolubility in electrolytes provide a distinctive platform for organizing redox‐active units within ion‐accessible frameworks, offering clear advantages over conventional molecular organic electrodes, which often suffer from dissolution, disordered packing, and poorly controlled transport pathways [9, 10, 11, 12, 13, 14].

Yet the practical performance of redox‐active COF cathodes still falls short of their conceptual promise. In many reported systems, the electrochemical response is limited not only by the intrinsic redox chemistry of the active motif, but also by incomplete utilization of buried sites, low intrinsic electronic conductivity, sluggish charge transport across stacked domains, and a strong dependence on electrode composition and electrolyte choice [15, 16, 17, 18, 19, 20, 21]. Accordingly, the field has moved beyond simply introducing electroactivity into a porous framework; the key challenge is now to establish structure–transport–electrolyte relationships that explain how framework design translates into practical battery performance [22, 23, 24].

One powerful route to expand the redox chemistry of COFs is post‐synthetic modification, in which electroactive units are covalently installed onto a pre‐formed crystalline scaffold [11, 25, 26, 27]. This strategy preserves the ordered porous architecture of the parent framework while avoiding the synthetic restrictions associated with directly constructing intrinsically redox‐active COFs from electroactive monomers. Among the available redox motifs, stable nitroxyl radicals such as 2,2,6,6‐tetramethylpiperidin‐1‐oxyl (TEMPO) are particularly attractive because they undergo a fast and reversible one‐electron oxidation from the nitroxyl radical to the oxoammonium cation [28, 29, 30]. Indeed, TEMPO‐based materials represent one of the most established families of p‐type organic electrodes, and the broader organic radical battery literature has been dominated by linear nitroxide polymers, especially PTMA‐type systems and related derivatives [31, 32, 33, 34]. From an electrochemical perspective, such materials are particularly interesting because they operate through a p‐type mechanism involving anion uptake. Compared with n‐type organic electrodes, p‐type systems generally access higher operating voltages, but their practical performance is often more strongly governed by the electrolyte, since the size, solvation structure, mobility, and interfacial reactivity of the counter‐anion can directly control redox‐site accessibility, rate capability, and cycling stability [35, 36, 37, 38, 39, 40]. For this reason, the development of TEMPO‐based COFs is particularly attractive since their behavior cannot be understood solely in terms of framework design; rather, it must be interpreted as the result of a coupled interaction between the radical‐bearing host and the electrolyte anion.

Importantly, however, the maturity of linear TEMPO‐polymer electrodes has not been translated into an equally developed family of porous TEMPO‐based polymers or COFs. This asymmetry is both significant and chemically meaningful. Linear nitroxide polymers are comparatively easy to synthesize, process, and integrate into composite electrodes, which has made them the dominant model systems for organic radical batteries [31, 32, 33, 34]. By contrast, porous TEMPO‐based frameworks must satisfy several demanding criteria simultaneously: the scaffold introduces electrochemically inactive mass that can dilute radical density; rigid porous networks often suffer from poor electronic percolation; post‐synthetic radical incorporation may be incomplete or spatially heterogeneous; and the benefits of porosity can only be realized when ion accessibility, framework integrity, and electrode‐level conductivity are all maintained under operating conditions [19, 20, 21, 22, 23, 24, 41, 42, 43]. Thus, despite the conceptual appeal of combining stable radical chemistry with ordered porous architectures, TEMPO‐based porous polymers remain far less mature than their linear counterparts. This gap is especially important because TEMPO‐functionalized porous frameworks should, in principle, provide a combination that linear systems cannot fully deliver: immobilized high‐voltage radical centers, structurally defined ion‐transport channels, and a programmable local environment for probing anion‐coupled charge storage [44, 45, 46, 47].

Although TEMPO‐functionalized COFs have previously been explored for electrochemical energy storage [16, 18, 48], and early studies have demonstrated their promise as high‐voltage organic electrodes [49], their application as high‐voltage cathode materials for Li‐ion batteries remains at an early stage. More importantly, systematic studies that disentangle the influence of framework structure from that of electrolyte anion chemistry are still scarce. This gap is non‐trivial: in high‐voltage radical‐based porous electrodes, apparent electrochemical improvements may arise not only from the design of the active framework, but also from differences in electrolyte stability window, anion transport, and cathode–electrolyte interfacial processes. A rigorous evaluation therefore requires more than material synthesis; it demands systematic comparison across electrolyte formulations to disentangle the intrinsic response of the radical‐functionalized framework from electrolyte‐driven effects [36, 37, 38, 39, 40, 50].

**SCHEME 1:**
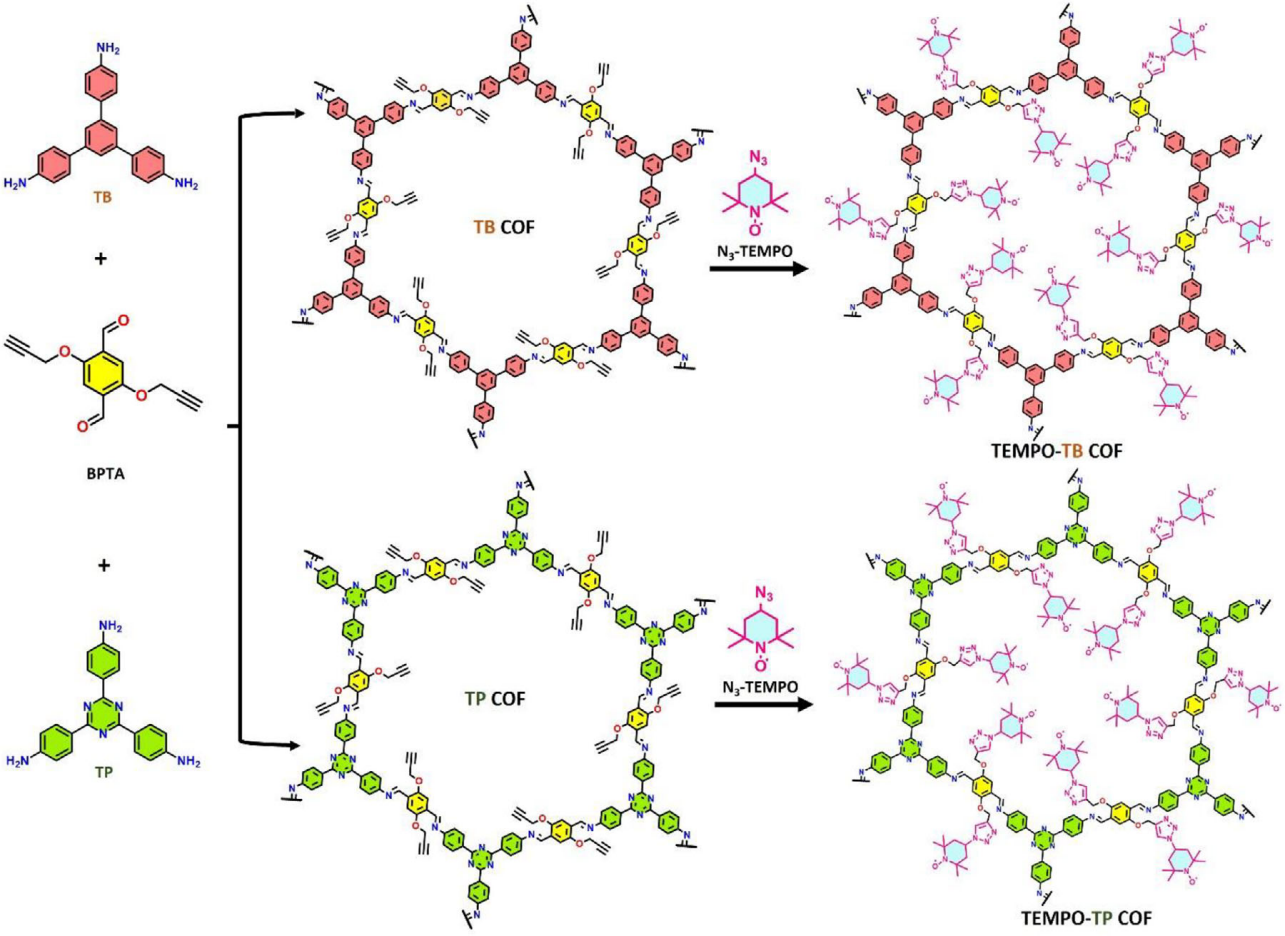
Schematic representation of the synthesis of **TEMPO‐TB** and **TEMPO‐TP** COFs.

In this work, we address this underdeveloped area by post‐synthetically modifying conjugated COF skeletons via click chemistry to graft 4‐azido‐2,2,6,6‐tetramethylpiperidine‐1‐oxyl radical (N_3_‐TEMPO) moieties onto the channel walls of two imine‐linked COFs, denoted **TB** and **TP** COFs (Scheme 1). Following synthesis and structural characterization of the parent frameworks, the resulting TEMPO‐functionalized materials (**TEMPO‐TB** and **TEMPO‐TP** COFs) were investigated as high‐voltage p‐type cathodes for Li‐organic batteries. The main structural difference between the two COFs lies in the presence of triazine moieties in **TEMPO‐TP**. Crucially, rather than treating the electrolyte as a secondary optimization parameter, we systematically examined the influence of lithium salt identity by comparing LiX electrolytes (X = PF_6_^−^, ClO_4_^−^, BF_4_^−^, DFOB^−^, and TFSI^−^) in carbonate‐based media. By correlating framework structure with salt‐dependent electrochemical response, this study elucidates how anion chemistry governs charge storage in TEMPO‐based porous radical cathodes and establishes framework–electrolyte matching as a decisive design principle for high‐voltage radical‐functionalized COF electrodes.

## 2 Results and Discussion

### 2.1 Synthesis and Structure Characterization of TEMPO‐COFs

To obtain the TEMPO‐COFs, first the two pristine COFs (**TB** and **TP** COFs) were synthesized under solvothermal conditions. The pristine COFs were synthesized by condensation reaction of 2,5‐bis(prop‐2‐yn‐1‐yloxy)terephthalaldehyde (BPTA) with the corresponding triamines (1,3,5‐tris(4‐aminophenyl)benzene (TB) or 2,4,6‐tris(4‐aminophenyl)‐1,3,5‐triazine (TP)) in a mixture of *ο*‐dichlorobenzene, *n*‐butanol, and 6 M aqueous acetic acid at 120°C for 72 h (see Supporting Information for experimental details). As shown in Scheme 1, the main structural difference between **TB** and **TP** COFs lies in the presence of triazine units in the **TP** COF. To incorporate TEMPO redox‐active functionality into the COF scaffolds, the pristine COFs were subjected to post‐synthetic modification with N_3_‐TEMPO radical via click chemistry (Scheme 1). The ethynyl groups present in the BPTA units are essential for this reaction, enabling a rapid azide‐alkyne cycloaddition with the azide moiety on the N_3_‐TEMPO radicals [18].

**FIGURE 1:**
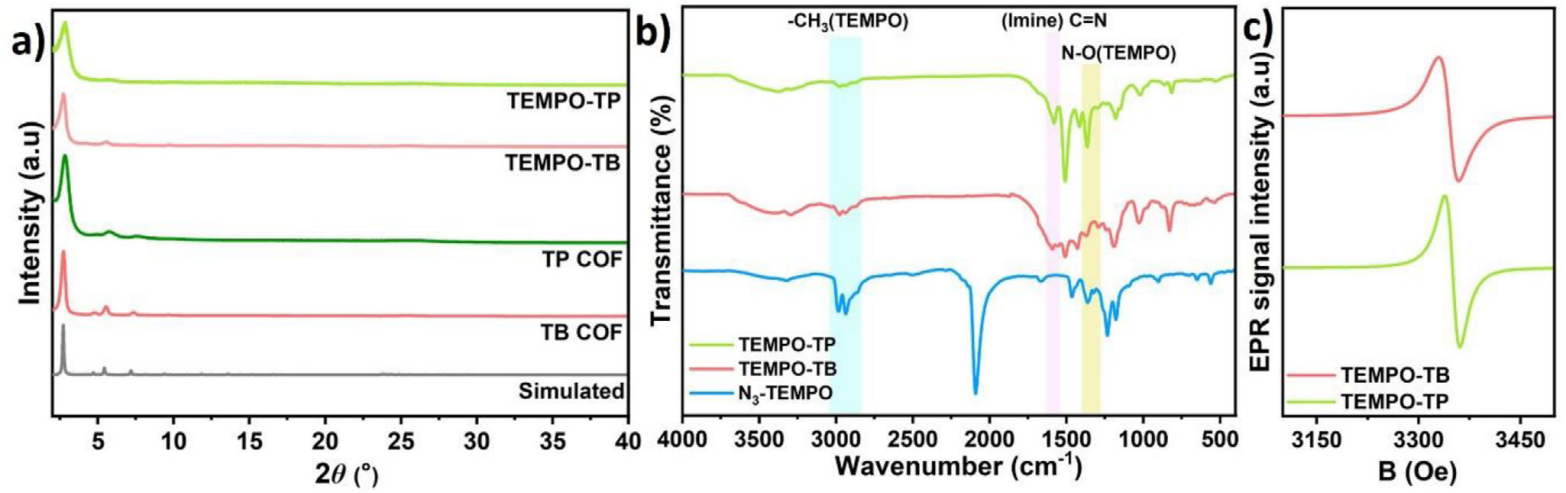
a) PXRD patterns of **TB**, **TP**, **TEMPO‐TB,** and **TEMPO‐TP** COFs and simulated PXRD pattern of **TB** COF. b) FT‐IR spectra of N_3_‐TEMPO radical, **TEMPO‐TB,** and **TEMPO‐TP** COFs. c) EPR spectra of **TEMPO‐TB** and **TEMPO‐TP** COFs at room temperature.

The successful synthesis of both pristine and TEMPO‐functionalized COFs was confirmed by powder X‐ray diffraction (PXRD). As shown in Figure 1a, an intense diffraction peak at 2.7° corresponding to the (100) plane was observed. The diffraction peaks closely matched the simulated PXRD pattern of the **TB** COF, which is isostructural to **TP** COF. After the click reaction, both TEMPO‐COFs retained their highly crystalline structure, as evidenced by their PXRD patterns. Fourier transform infrared (FT‐IR) spectra of **TB** COF and **TP** COF (Figure 1b and Figures S1 and S2) further confirm successful framework formation. The disappearance of the aldehyde (C═O) stretching band at ∼1688 cm^−1^ and the amine (N─H) band at 3300−3400 cm^−1^, along with the appearance of a new band at 1592 cm^−1^, verified the formation of imine (C═N) linkages via the Schiff‐base condensation. Following the click reaction, the stretching vibrations in the 2930–3000 cm^−1^ region, assigned to the ethynyl functional groups on the BPTA units, decreased in intensity, confirming covalent attachment of the N_3_‐TEMPO radical. The methyl stretching bands (2938 and 2985 cm^−1^) and N─O stretching band (1368 cm^−1^) associated with the TEMPO radical remained intact, indicating successful incorporation of the redox‐active moiety [16, 18].

Electron paramagnetic resonance (EPR) further confirmed the radical nature of the TEMPO‐COFs (Figure 1c). The solid‐state EPR spectra of TEMPO‐COFs revealed a single signal at *g* = 2.016 and 2.014 for **TEMPO‐TB** and **TEMPO‐TP**, respectively, consistent with the presence of TEMPO radicals within the COFs. Quantitative EPR analysis revealed that the number of incorporated TEMPO radicals per unit cell was 4.5 and 4.2 molecules for **TEMPO‐TB** and **TEMPO‐TP**, respectively (see SI).

The morphology of both the pristine and TEMPO‐functionalized COFs was examined using scanning electron microscopy (SEM). As shown in Figures S3 and S4, all materials exhibit an irregular, spherical‐like morphology that is largely preserved after TEMPO functionalization, indicating that the post‐synthetic modification does not significantly disrupt the overall microstructure. High‐resolution transmission electron microscopy (HRTEM) images of TEMPO‐COFs reveal well‐defined periodic lattice fringes extending across large domains, providing real‐space confirmation of the high degree of crystallinity indicated by the intense peaks in the PXRD patterns (Figure S5).

Because ion transport and charge storage are strongly influenced by porosity, N_2_ sorption measurements were performed at 77 K. Both COFs displayed Type‐I isotherms, characteristic of microporous materials. The BET surface areas of **TB** and **TP** COFs were calculated to be 1434 and 932 m^2^g^−1^, respectively. After the click reaction, the surface areas decreased to 644 and 499 m^2^g^−1^ for **TEMPO‐TB** and **TEMPO‐TP**, respectively (Figures S6 and S7). The pore size distribution analysis also confirmed the reduction in pore size for TEMPO‐COFs (Figures S8 and S9). The reduction in porosity is attributed to the presence of flexible TEMPO radical group covalently anchored within the pores, though sufficient porosity remains to enable ion accessibility. The well‐defined microporosity is expected to promote rapid ion mobility and efficient electrolyte infiltration, supporting stable electrochemical cycling. Thermogravimetric analysis (TGA) (Figures S10 and S11) revealed that the pristine COFs are thermally stable up to 300°C, while the TEMPO‐COFs exhibit stability up to 220°C. This decrease in thermal stability is attributed to the presence of the TEMPO moieties, which are more thermally labile than the imine‐linked COF backbone. The TGA profiles of TEMPO‐COFs display a two‐step decomposition behavior, with an initial weight loss in the intermediate temperature range (∼200°C–350°C) assigned to the degradation of grafted TEMPO groups, followed by decomposition of the COF framework at higher temperatures. Overall, the TEMPO‐COFs retain sufficient thermal robustness for battery operation under typical conditions, and the intermediate‐temperature weight loss further supports the successful incorporation of TEMPO radicals within the framework.

### 2.2 Electrochemical Performance of TEMPO‐COF Buckypaper Electrodes in Li Half‐Cells

#### 2.2.1 Electrolyte‐Dependent CV and GCD Response

To evaluate the electrochemical properties of TEMPO‐COFs and their potential use in non‐aqueous organic batteries, buckypaper electrodes were fabricated, assembled in Li half‐cells, and subsequently tested electrochemically (see Supporting Information for experimental details). These self‐standing, binder‐ and metal current collector‐free buckypaper electrodes minimize inactive mass while enabling high‐mass‐loadings and improved electrochemical performance [14, 43]. This strategy, previously applied to classical redox‐active polymers, was extended by our group to COFs, with our recent TTF‐COF study representing one of the few examples of COF‐based buckypaper cathodes for Li‐ion half‐cells [14].

For initial electrochemical screening, TEMPO‐COF buckypapers were fabricated with a mass loading of 2 mg cm^−^^2^ and a composition of 60:30:10 wt.% (TEMPO‐COF:SWCNT:rGO wt.%). Because TEMPO‐based electrodes operate primarily via a p‐type mechanism at high potentials, oxidation of the nitroxide radical to the corresponding oxoammonium cation (TEMPO^•^/TEMPO^+^) during charging is compensated by electrolyte anion uptake rather than Li^+^ insertion. Consequently, the nature, size, and mobility of the anion can strongly influence the redox potential, polarization, rate capability, and cycling stability of the electrode. Motivated by this anion‐coupled charge‐storage mechanism, we systematically investigated a series of carbonate‐based electrolytes containing 1 M lithium salts (LiPF_6_, LiClO_4_, LiBF_4_, LiDFOB, or LiTFSI) in EC/DMC (3:7, v/v).

**FIGURE 2:**
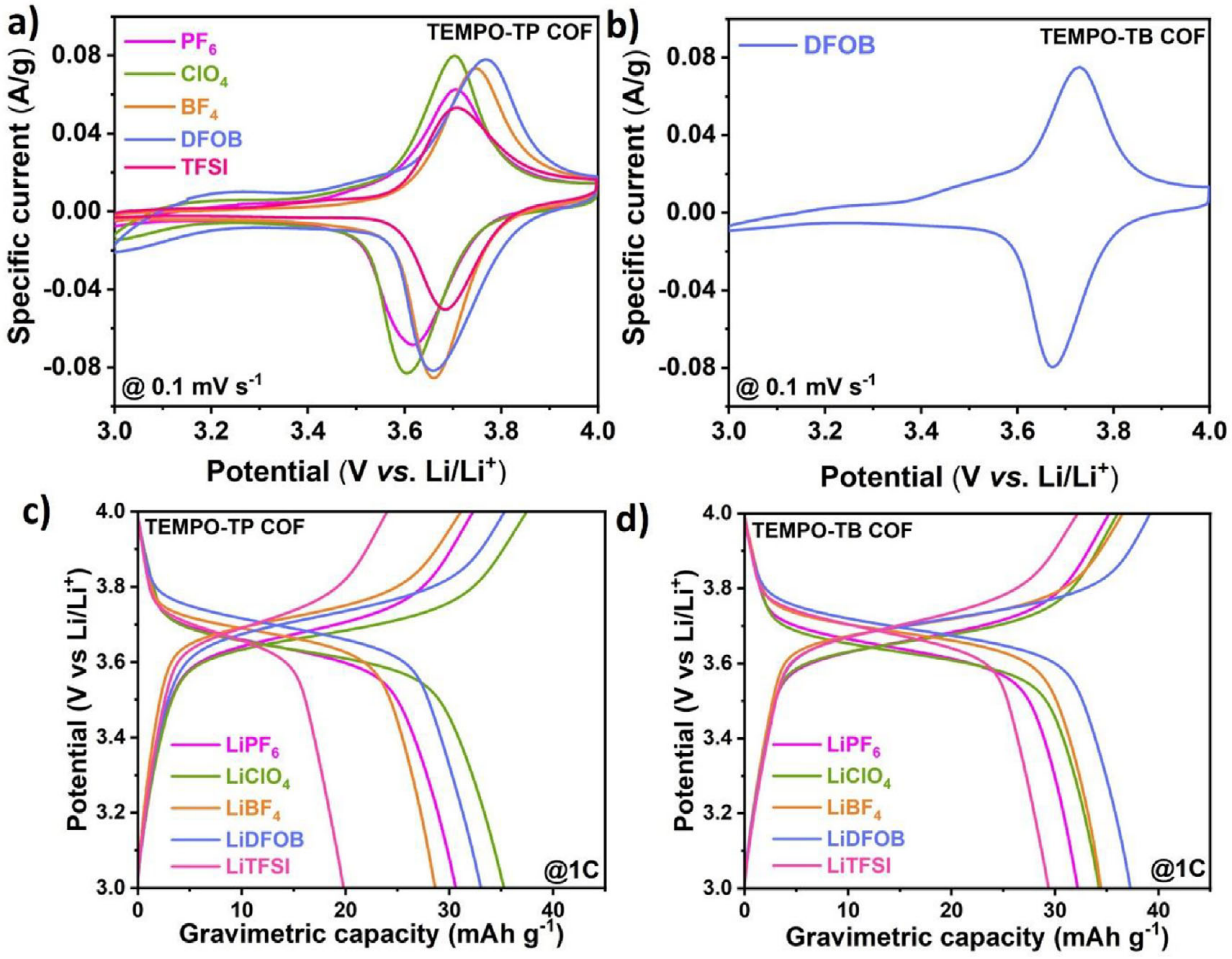
Electrolyte‐dependent CV and galvanostatic response of TEMPO‐COF buckypaper electrodes in Li half‐cells. (a, b) CVs of the two TEMPO‐COF electrodes recorded at 0.1 mV s^−^^1^ in 1 M LiX dissolved in EC/DMC (3:7, v/v), where X = PF_6_^−^, ClO_4_^−^, BF_4_^−^, DFOB^−^, or TFSI^−^. (c, d) Corresponding GCD profiles at 1 C for the same electrodes in the selected electrolytes. Electrode active mass loading ∼2 mg cm^−^^2^. Electrode composition 60:30:10 wt.%.

First, cyclic voltametric (CV) behavior of both TEMPO‐COFs was examined in the high‐potential window of 3.0–4.0 V vs. Li/Li^+^, where TEMPO‐based electrodes are known to display their most reversible p‐type redox chemistry (TEMPO^•^/TEMPO^+^). In this regime, oxidation of the nitroxide radical to the corresponding oxoammonium species is charge‐balanced by electrolyte‐anion uptake, while the reverse cathodic sweep restores the neutral TEMPO state. Accordingly, CV was used to compare the intrinsic redox response of the **TEMPO‐TP** COF buckypaper electrodes in different electrolytes and to assess the extent to which electrolyte composition influences peak position, polarization, and reversibility. Whereas in the case of **TEMPO‐TB** COF, the CV was measured in LiDFOB electrolyte only. Figure 2a shows that **TEMPO‐TP** COF retains a single dominant TEMPO‐centered redox couple in all electrolytes, while both the formal potential and the polarization remain clearly anion‐dependent. From the CV traces of **TEMPO‐TP** at 0.1 mV/s (Figure 2a), the anodic/cathodic peak positions are estimated to be at 3.77/3.66 V for LiClO_4_, 3.77/3.67 V for LiPF_6_, 3.82/3.72 V for LiBF_4_, 3.84/3.72 V for LiDFOB, and 3.78/3.75 V for LiTFSI, corresponding to *E*_1/2_ values of approximately 3.72, 3.72, 3.77, 3.78, and 3.76 V, and peak separations (Δ*E*_p_) of about 0.11, 0.10, 0.10, 0.12, and 0.03 V, respectively. Thus, ClO_4_^−^ and PF_6_^−^ place the TEMPO^•^/TEMPO^+^ couple at comparatively lower potentials, whereas BF_4_^−^ and especially DFOB^−^ shift the redox process anodically; among these electrolytes, DFOB^−^ shows the largest polarization. The unusually small apparent Δ*E*_p_ for TFSI^−^ should, however, be interpreted cautiously, since its voltammetric response is broader and less intense. Overall, these results confirm that the redox behavior of the TEMPO‐COFs is strongly governed by the identity of the compensating anion, in line with the established anion‐coupled p‐type charge‐storage mechanism of TEMPO‐based electrodes [29, 46]. For **TEMPO‐TB** in LiDFOB‐containing electrolyte (Figure 2b), the CV displays a single sharp TEMPO‐centered redox couple at 3.73/3.67 V vs. Li/Li^+^ (*E*_1/2_ ≈ 3.70 V; Δ*E*_p_ ≈ 0.06 V), indicating a highly reversible p‐type process with lower polarization than that observed for **TEMPO‐TP** using the same anion.

The galvanostatic profiles at 1 C further support the electrolyte‐dependent trends observed by CV (Figure 2c,d). From the GCD curves for **TEMPO‐TP**, the average discharge potentials are approximately 3.63, 3.64, 3.67, 3.68, and 3.66 V for LiPF_6_, LiClO_4_, LiBF_4_, LiDFOB, and LiTFSI, respectively (Figure 2c). The corresponding apparent charge–discharge polarizations are about 0.10, 0.10, 0.08, 0.09, and 0.06 V, while the 1 C discharge capacities are ca. 31, 35, 27, 31, and 20 mAh g^−^^1^, respectively. The GCD profiles of TEMPO‐TB likewise confirm the electrolyte‐dependent behavior inferred from CV, with approximate average discharge potentials of 3.62 V (LiPF_6_), 3.64 V (LiClO_4_), 3.67 V (LiBF_4_), 3.68 V (LiDFOB), and 3.66 V (LiTFSI) (Figure 2d). The corresponding apparent charge–discharge polarizations are 0.09, 0.08, 0.06, 0.07, and 0.07 V, respectively, while the 1 C discharge capacities are about ∼30, ∼34, ∼28, ∼33, and ∼20 mAh g^−^^1^.

As mentioned above, TEMPO‐based electrodes are known to exhibit their most reversible electrochemical response through the p‐type mechanism at higher potentials, whereas the n‐type reduction (TEMPO^•^/TEMPO^−^) occurring at lower potentials is typically far less reversible and more prone to parasitic reactions in conventional carbonate electrolytes [19, 44]. To further probe the potential n‐type activity of the TEMPO functionalities, a glyme‐based electrolyte (1 M LiTFSI in TEGDME) being generally more suitable for probing reduction processes, was employed to evaluate the performance of TEMPO‐COFs at lower potentials (Figures S12 and S13 and discussion in ESI) [21, 45]. Overall, both TEMPO‐COF electrodes displayed poorly reversible low‐potential redox signatures, indicating that the accessibility to additional charge from n‐type redox process is governed not only by solvent stability, but also by the coupled effects of anion/solvation chemistry and electrode–electrolyte interfacial processes [19, 28].

#### 2.2.2 Electrolyte‐Dependent Rate Capability

**FIGURE 3:**
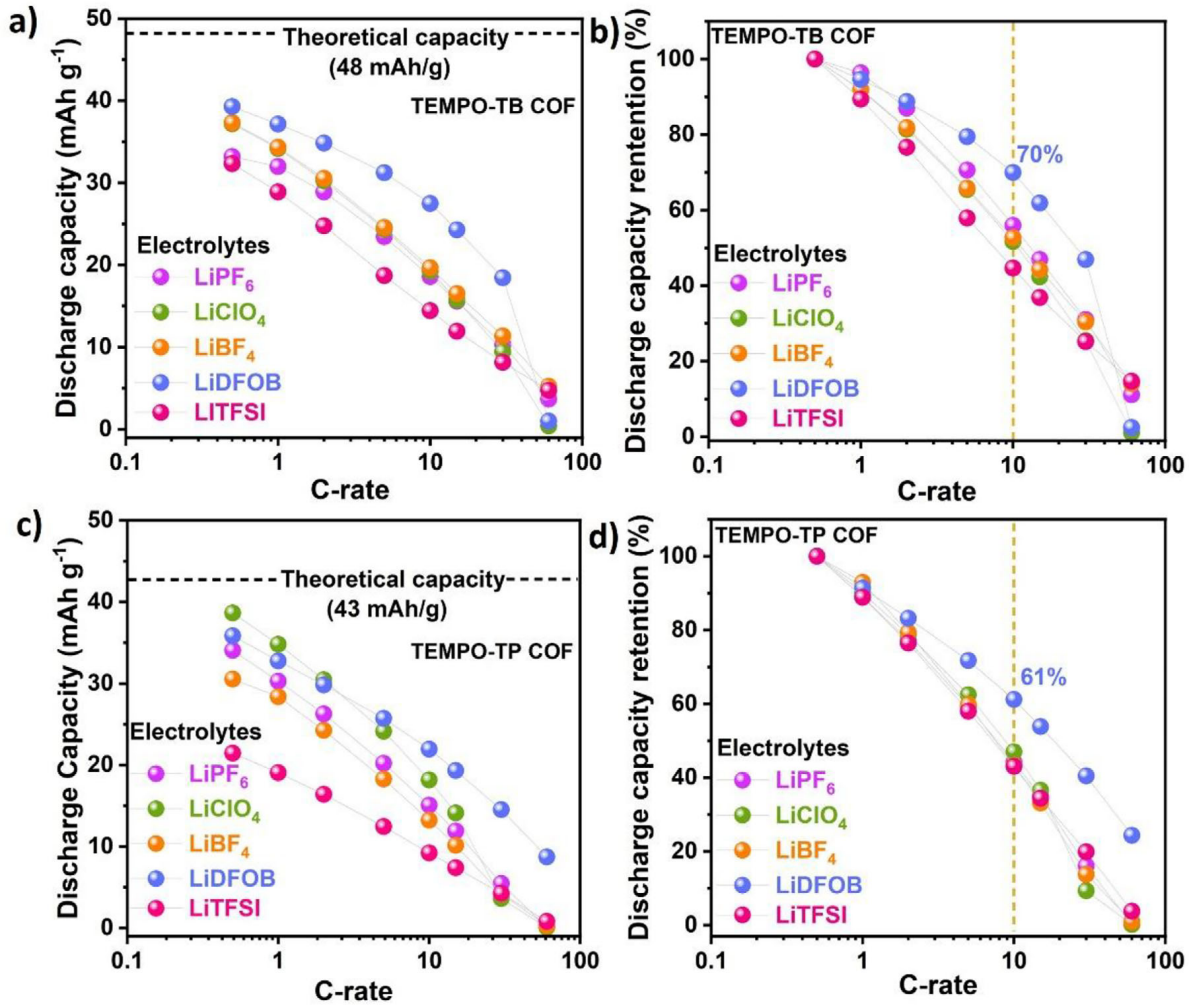
Rate performance of TEMPO‐COF buckypaper electrodes in Li half‐cells in different electrolytes. (a, c) Discharge capacity vs. C‐rates of **TEMPO‐TB** (a) and **TEMPO‐TP** (c). (b, d) discharge capacity retention vs. C‐rates of **TEMPO‐TB** (b) and **TEMPO‐TP** (d). The discharge capacities at higher C‐rates were normalized with respect to the discharge capacity at 0.5 C. Electrode active mass loading ∼2 mg cm^−^^2^. Electrode composition 60:30:10 wt.%.

The electrolyte‐dependent rate capability of the two TEMPO‐COF electrodes was examined by galvanostatic cycling at increasing C‐rates (Figure 3). While TEMPO‐based electrodes are generally associated with fast p‐type redox kinetics, the present data clearly show that the reversible capacity retained at high rates is strongly influenced by the electrolyte anion. Because the TEMPO redox process is charge‐balanced by anion uptake and release, electrolyte composition can directly govern polarization, charge‐transfer, ion transport, and the effective utilization of redox‐active sites [28, 29]. Similar trends have been reported previously for TEMPO‐based polymer electrodes, albeit mainly in aqueous media [35, 47].

Based on the one‐electron TEMPO^•^/TEMPO^+^ process, the theoretical specific capacities of **TEMPO‐TB** and **TEMPO‐TP** COFs were calculated to be 48.5 and 43.7 mA h g^−^^1^, respectively (see SI). As shown in Figure 3a,c, both COFs delivered their highest low‐rate capacities in LiClO_4_ or LiDFOB‐based electrolytes, whereas LiTFSI consistently resulted in the lowest discharge capacities. At 0.5 C, TEMPO‐TB reached 39.3 mA h g^−^^1^ in LiDFOB, while **TEMPO‐TP** delivered 38.7 mA h g^−^^1^ in LiClO_4_ and 35.9 mA h g^−^^1^ in LiDFOB. Upon increasing the rate, the capacity of both frameworks decreased progressively across all electrolytes; however, the decay was least pronounced for LiDFOB. This trend is clearly reflected in the discharge‐capacity retention plots (Figure 3b,d), where LiDFOB provides the highest retention at 10 C for both COFs, remaining around 70% and 61% for **TEMPO‐TB** and **TEMPO‐TP**, respectively.

The cycle‐resolved rate test (Figure S14) and the corresponding Coulombic efficiency profiles (Figure S15) further support this conclusion. In both COFs, LiDFOB shows the smallest stepwise capacity loss upon increasing current and among the most stable Coulombic efficiencies across the full rate sequence. The substantial recovery of capacity upon returning to the initial low C‐rate indicates that the performance loss at high rates is predominantly kinetic rather than irreversible, confirming the structural robustness and reversible redox activity of the TEMPO‐COF frameworks.

The galvanostatic profiles (Figures S16 and S17) remain centered around the characteristic TEMPO plateau at ∼3.6–3.7 V vs. Li/Li^+^, but the voltage polarization becomes increasingly electrolyte‐dependent at higher currents. At 1 C, LiClO_4_ and LiDFOB provide the best balance of average discharge potential, moderate polarization, and practical capacity in both frameworks, whereas LiTFSI gives markedly lower capacities, indicating less effective utilization of the TEMPO redox sites under galvanostatic conditions.

This framework‐dependent but overall superior behavior of LiDFOB is particularly evident in the capacity‐utilization analysis (Figure S18). At 10 C, the utilization reaches 56% for **TEMPO‐TB** and 50% for **TEMPO‐TP** in LiDFOB, compared with only 29% and 21%, respectively, in LiTFSI. Taken together, these results indicate that although both TEMPO‐COFs retain the fast redox character expected for nitroxide‐based cathodes, the electrolyte anion plays a decisive role in determining how effectively that intrinsic kinetics can be translated into practical high‐rate performance. In this regard, LiDFOB emerges as the most favorable overall electrolyte.

#### 2.2.3 Kinetic Origin of the Electrolyte‐Dependent Rate Performance

Given the similar electrochemical trends observed for both COFs across the electrolyte series, the kinetic analysis was focused on the **TEMPO‐TP** COF as a representative system, allowing a more detailed and systematic investigation.

**FIGURE 4:**
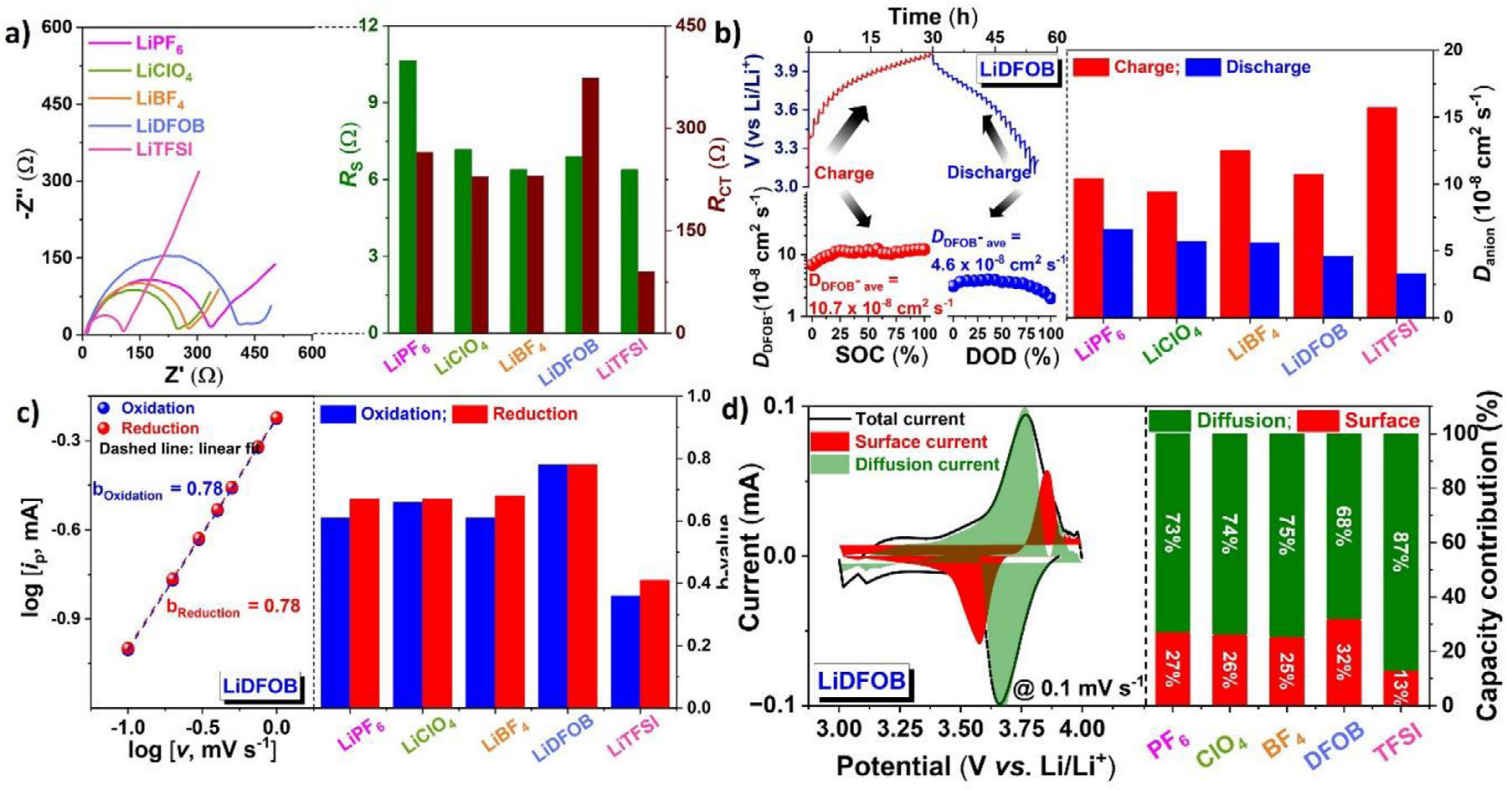
Electrochemical kinetics evaluation of **TEMPO‐TP** COF buckypaper electrode in Li half‐cell in different electrolytes. (a) Representative Nyquist plots at equilibrium charging potential of ∼3.65 V (approx. 50% SoC) vs. Li/Li^+^, and the corresponding *R*_s_ and *R*_ct_ values. (b) *D*_anion_ diffusivity as a function of SoC/DoD (bottom panel) calculated from GITT measurement (top panel) for the representative LiDFOB (left). The *D*_anion_ in different electrolytes is shown on the right. (c) Peak current vs. scan rate in logarithmic scale to obtain *b*‐values according to *i*_p_ = a*v* ^b^ for the representative LiDFOB electrolyte (left). The *b*‐value for different electrolytes is shown on the right. d) Surface‐ and diffusion‐controlled current contributions in CV for the representative LiDFOB electrolyte at 0.1 mV s^−1^ (left). Surface‐ and diffusion‐capacity contributions calculated applying the Dunn method in different electrolytes at 0.1 mV s^−1^ is shown on the right. Electrode active mass loading ∼2 mg cm^−^^2^. Electrode composition 60:30:10 wt.%.

To clarify the origin of the electrolyte‐dependent rate performance, the redox kinetics of **TEMPO‐TP** COF electrodes were subsequently investigated by potentiostatic electrochemical impedance spectroscopy (EIS), galvanostatic intermittent titration technique (GITT), and scan‐rate‐dependent CV and quantitative kinetic analysis (Figure 4). EIS was performed at various potentials during both charging and discharging (Figure S19). All Nyquist plots displayed a depressed semicircle at high frequencies and a sloped line at low frequencies (Figure 4a and Figures S20–S24), indicative of charge‐transfer and diffusion processes [37]. Before comparing the electrolytes at a fixed state of charge, it is useful to note the general potential dependence of the impedance response.

As shown in Figure S25, equivalent series resistance (*R*_s_), determined from the high‐frequency intercept on the Z´ axis, changes only weakly with potential across all electrolytes, indicating that the ohmic contribution is governed mainly by the electrolyte, separator, and overall cell configuration, rather than by the redox state of the TEMPO‐COF electrode. In contrast, charge‐transfer resistance (*R*_ct_), estimated from the diameter of the semicircle, exhibits a clear dependence on state of charge: it decreases from the initial charging potential to a minimum within the main TEMPO redox region, and increases again upon further discharge to lower potentials. This behavior suggests that charge‐transfer kinetics are most favorable within the principal TEMPO^•^/TEMPO^+^ conversion window, where partial oxidation likely promotes more efficient electron hopping within the redox‐active framework while simultaneously facilitating anion exchange and desolvation at the interface [38, 39]. By contrast, at the beginning of charge and especially at low potentials during deep discharge, the system exhibits higher R_ct_ values, consistent with lower effective charge‐carrier density, less favorable interfacial ion‐transfer processes, and possibly greater interfacial heterogeneity or passivation. Overall, while *R*_s_ remains largely insensitive to state of charge, the variation in *R*_ct_ provides a direct measure of how the redox state and electrolyte environment together govern the interfacial kinetics of the TEMPO‐COF electrodes.

At the representative state‐of‐charge (SoC ≈ 50%) selected for comparison (Figure 4a), the impedance response is strongly electrolyte‐dependent. *R*_s_ varies only modestly across the electrolytes, with LiPF_6_ showing the highest ohmic resistance (≈10 Ω range) and the other electrolytes showing similar, slightly lower values (ranging 6−8 Ω). In contrast, *R*_ct_ differs much more markedly. LiTFSI exhibits the smallest semicircle and the lowest *R*_ct_, LiPF_6_, LiClO_4_, and LiBF_4_ show intermediate values, whereas LiDFOB displays larger interfacial resistance. In summary, these results indicate that the electrolyte identity influences TEMPO‐COF performance primarily through changes in interfacial redox kinetics rather than bulk ohmic resistance.

GITT measurements of **TEMPO‐TP** COF further revealed a pronounced electrolyte dependence of the apparent anion diffusivity (Figure 4b and Figures S26–S30). In all cases, *D*_anion_ remained on the order of 10^−^^8^ cm^2^ s^−^^1^, but the average values during charging were consistently higher than during discharging. The charge‐side diffusivities followed the order TFSI^−^ (15.7) > BF_4_^−^ (12.5) > DFOB^−^ (10.7) ≈ PF_6_^−^ (10.4) > ClO_4_^−^ (9.4) × 10^−^^8^ cm^2^ s^−^^1^, whereas the discharge‐side values decreased to 6.6, 5.7, 5.6, 4.6, and 3.3 × 10^−^^8^ cm^2^ s^−^^1^ for TFSI^−^, BF_4_^−^, DFOB^−^, PF_6_^−^, and ClO_4_^−^ respectively. In general, *D*_anion_ either increased slightly or remained nearly constant with SoC during charging, while lower and flatter values were observed during discharge, often followed by a decline toward deep discharge. This behavior suggests that anion transport is more favorable in the principal TEMPO oxidation window, where the growing population of oxoammonium sites promotes anion compensation and transport through the porous framework [40]. By contrast, during discharge, the progressive loss of oxidized TEMPO sites, together with less favorable anion deinsertion and possible transient trapping or redistribution within the pores, leads to lower apparent diffusivity.

Taken together, the EIS and GITT results provide a consistent picture of how the TEMPO‐COF electrodes evolve with potential. Their common evolution with potential supports the view that the principal TEMPO oxidation window is the kinetically most favorable region for both charge transfer and anion transport, whereas the terminal states are limited by slower interfacial exchange and less efficient anion transport. Additionally, supported by the prior PTMA‐based literature [48, 49], these results indicate that practical high‐rate performance is governed not by a single kinetic descriptor, but by the interplay of bulk anion transport, interfacial charge‐transfer, reversible anion compensation, and electrolyte–electrode compatibility; accordingly, the superior diffusivity and low *R*_ct_ observed for LiTFSI are not by themselves sufficient to ensure the best electrochemical response.

Finally, CV at scan rates between 0.1 and 1.0 mV s^−^^1^ (Figure S31) showed that, except for LiTFSI, the **TEMPO‐TP** electrode retained a clear and well‐defined redox couple with increasing scan rate, despite the expected peak shift and broadening, indicating comparatively reversible and kinetically facile redox behavior. The charge‐storage mechanism was further assessed by correlating peak current (*i_p_*) with scan rate (ν) through the power‐law expression *i_p_ = a·v^b^*, obtained from the slope of the $\log(i_{p})$ v log(ν) plots (Figure 4c and Figure S32). In general, *b* ≈ 0.5 denotes diffusion‐controlled behavior, whereas *b* ≈ 1 is characteristic of a capacitive‐ or surface‐controlled response; values between these limits indicate mixed kinetics.

The extracted *b*‐values reveal a clear electrolyte dependence of the redox kinetics: LiDFOB gives the highest values for both oxidation and reduction (*b*_ox_ =  0.78, *b*_red_ =  0.78), indicating the largest pseudocapacitive or surface‐controlled contribution among the electrolytes tested. LiClO_4_ (0.67/0.66), LiBF_4_ (0.68/0.61), and LiPF_6_ (0.67/0.61) show intermediate *b*‐values, consistent with a mixed mechanism in which both diffusion‐controlled and capacitive processes contribute appreciably. By contrast, LiTFSI exhibits markedly lower values (0.36/0.41), pointing to a much stronger diffusion‐limited character. Dunn's analysis confirms that the charge‐storage mechanism of TEMPO‐TP is strongly electrolyte‐dependent (Figure 4d and Figures S33–S38). At 0.1 mV s^−^^1^, diffusion‐controlled current dominates in all electrolytes, but LiDFOB already shows the highest surface‐controlled contribution (32%), compared with 25%–27% for LiPF_6_, LiClO_4_, and LiBF_4_, and only 13% for LiTFSI. With increasing scan rate, the surface contribution rises for all systems, reaching 60% in LiDFOB at 1.0 mV s^−^^1^, whereas LiTFSI remains largely diffusion‐controlled (32% surface contribution) (Figure 4d). This trend agrees well with the *b*‐values and rate‐capability data, indicating that LiDFOB promotes the most favorable pseudocapacitive response, while LiTFSI suffers from the weakest practical charge utilization.

Overall, these results indicate that the most favorable high‐rate response is obtained when the **TEMPO‐TP** framework combines rapid anion‐coupled redox with a substantial pseudocapacitive contribution, as in LiDFOB, whereas the LiTFSI electrolyte leads to kinetically less favorable charge storage despite its low *R_ct_* and high charge‐side diffusivity. This interpretation is consistent with prior studies on redox‐active polymers, where higher *b*‐values generally correlate with faster charge delivery, greater capacitive dominance, and improved rate capability.

#### 2.2.4 Electrolyte‐Dependent Cycle Stability

**FIGURE 5:**
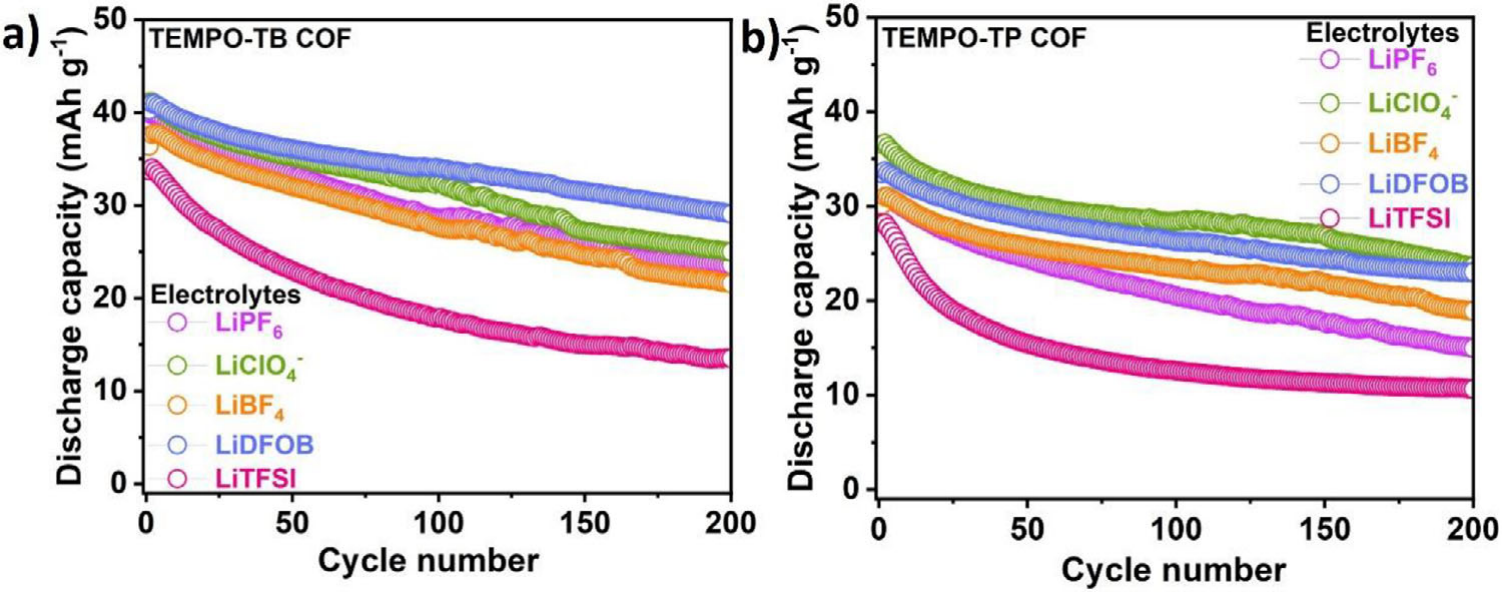
Cycle stability: discharge capacity vs. cycle number of **TEMPO‐TB COF** (a) and **TEMPO‐TP COF** (b) in different electrolytes recorded at 1 C. Electrode active mass loading ∼2 mg cm^−^^2^. Electrode composition 60:30:10 wt.%.

The long‐term cycling stability of the two TEMPO‐COF electrodes was evaluated in Li half‐cells at 1 C using the five electrolytes investigated (Figure 5 and Figures S39–S41 and Table S1). TEMPO‐TB COF delivered initial capacities of 39.7, 41.1, 37.6, 40.9, and 34.0 mA h g^−^^1^ in LiPF_6_, LiClO_4_, LiBF_4_, LiDFOB, and LiTFSI, respectively, while TEMPO‐TP showed initial capacities of 31.0, 36.7, 32.8, 36.4, and 22.4 mAh g^−^^1^ under the same conditions. LiDFOB afforded the best cyclability for both electrodes, retaining 72.1% and 68.7% of the initial capacity after 200 cycles for TEMPO‐TB and TEMPO‐TP, respectively, whereas LiTFSI gave the poorest retention (40% and 37.7%). The representative GCD profiles (Figures S39–S40) further show that LiDFOB better preserves the characteristic TEMPO plateau and limits polarization growth upon cycling, while LiTFSI undergoes a much stronger shrinkage of accessible capacity. After a short formation period, the Coulombic efficiency remained close to 100% in both TEMPO‐COFs, indicating largely reversible redox chemistry across all electrolytes (Figure S41). Taken together with the kinetic analysis, these results indicate that the superior cycling stability in LiDFOB arises from a more favorable balance of interfacial compatibility, anion transport, and charge‐transfer kinetics.

#### 2.2.5 TEMPO‐TP COF Electrodes With High‐Mass‐Loading and High‐Active‐Material‐Content

For practical LIBs, increasing the active‐material fraction and electrode mass loading is essential because the cell‐level energy density and cost are strongly penalized when thin electrodes are used and inactive components dominate the electrode stack [45, 50, 51]. This challenge is particularly acute for organic cathodes, where low electronic conductivity often forces the use of thin coatings and large carbon content [49, 52]. In the specific case of TEMPO/PTMA‐type materials, to the best of our knowledge, most studies still operate in the low‐loading regime (< 2 mg cm^−^^2^), while the most practically oriented PTMA systems report active mass loadings only up to 9.65 mg cm^−^^2^ [52].

In this context, given the similar electrochemical behavior of both COFs, the high‐mass‐loading and high‐active‐material‐content study was carried out using the **TEMPO‐TP** COF as a representative system and was limited to the LiDFOB electrolyte, which showed the most favorable overall performance. We investigated the high‐mass‐loading performance of the **TEMPO‐TP** buckypaper electrode by increasing the active‐material loading from the standard low‐loading condition (∼2 mg cm^−^^2^) to 5, 10, 20, and 40 mg cm^−^^2^. Moreover, to further enhance the practical relevance of the electrodes, the active‐material content was increased from 60 wt.% (60:30:10, TEMPO‐COF:SWCNT:rGO) to 80 wt.% (80:15:5, TEMPO‐COF:SWCNT:rGO), thereby reducing the fraction of inactive carbon components from 40 wt.% to only 20 wt.%.

**FIGURE 6:**
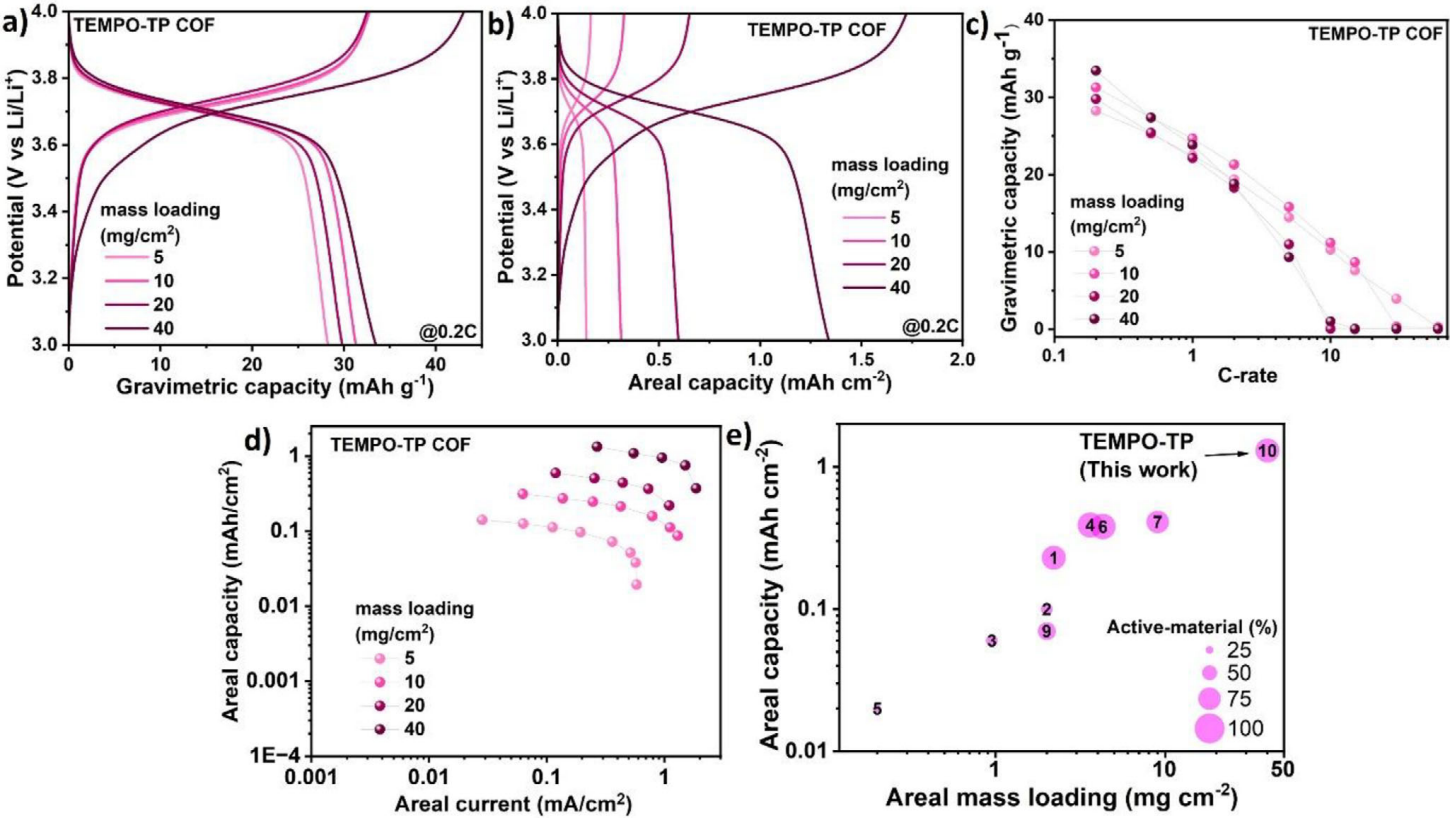
Gravimetric/areal performance evaluation of **TEMPO‐TP COF** electrodes buckypaper electrodes with different mass loadings in Li half‐cells. Electrode composition 80:15:5 wt.%. (a) GCD gravimetric capacity‐potential profiles and areal capacity‐potential profiles (b). (c) Gravimetric capacity vs. C‐rate. (d) Areal capacities as a function of areal current. (e) Comparing areal capacity of **TEMPO‐TP COF** electrode with state‐of‐the‐art TEMPO/PTMA cathodes. This figure is computed by considering some of the best‐performing TEMPO/PTMA‐based organic electrodes in Li‐cells (see Table S2).

At 0.2 C, the voltage profiles remain well defined, and the increase in polarization with loading is only moderate (Figure 6a,b). Importantly, the gravimetric capacity is retained within a relatively narrow range of approximately 30–35 mAh g^−^^1^ across the entire loading range (Figure 6a), indicating that the thicker electrodes still preserve a substantial fraction of the accessible TEMPO redox sites. As a result, Figure 6b shows that the areal capacity increases approximately proportionally with mass loading, reaching about 1.3 mAh cm^−^^2^ with high mass loading electrodes (40 mg cm^−^^2^). At higher C‐rates, however, the expected loading penalty becomes more evident: the gravimetric capacity decreases progressively with both increasing rate and electrode thickness, with the most significant deterioration observed for the 40 mg cm^−^^2^ electrode (Figure 6c and Figure S42). Nevertheless, even at a high areal current of 3.5 mA/cm^2^ (corresponding to 2 C), the areal capacity of the 40 mg cm^−^^2^ electrode remains above 0.7 mAh/cm^2^ (Figure 6d). Additionally, the Coulombic efficiency also remains close to 100%, indicating that the thick electrodes maintain stable and reversible electrochemical behavior under these conditions (Figure S42a).

To further assess the practical relevance of the **TEMPO‐TP COF** cathode, cycling tests were performed at progressively increased mass loadings of 5, 10, 20, and 40 mg cm^−^^2^ in LiDFOB electrolyte at 1 C (Figures S43 and S44). As expected, increasing the electrode mass loading led to a marked improvement in areal capacity, with the 40 mg cm^−^^2^ electrode delivering an initial areal capacity of ca. 1.25 mAh cm^−^^2^ at 1 C and retaining approximately 0.97 mAh cm^−^^2^ after 50 cycles (Figure S43a). Despite the use of thick buckypaper electrodes, the Coulombic efficiency rapidly approached ∼100% after the initial cycles, indicating highly reversible TEMPO redox chemistry under high‐loading conditions (Figure S43b). The capacity retention remained in the range of ∼77%–82% after 50 cycles for all mass loadings, reflecting the increasing transport and polarization limitations associated with thick organic electrodes while still demonstrating stable cycling at practically relevant areal capacities (Figure S43c). Representative GCD profiles further confirm the preservation of the characteristic TEMPO redox plateau around 3.6–3.8 V vs. Li/Li^+^ during cycling, including for high‐mass‐loading electrodes (Figure S44).

From a state‐of‐the‐art perspective, these results are noteworthy for a TEMPO‐based Li‐ion cathode. Although the areal capacities reported here are still below 3 mAh/cm^2^ – the current benchmark for practical LIBs, the present system operates at a much lower intrinsic specific capacity. Therefore, the relevant metric is not the absolute areal capacity alone, but rather the ability to retain meaningful gravimetric performance at very high electrode thickness and high active‐material fraction. In this context, extending the TEMPO‐COF electrode to 40 mg cm^−^^2^ at 80 wt.% active content substantially surpasses the loading regime usually explored for TEMPO/PTMA materials. The resulting areal capacity of ∼1.3 mAh cm^−^^2^ obtained at 0.2 C is already competitive with, or even exceeds, values reported for practical PTMA electrodes, despite the substantially more demanding loading conditions (Figure 6e and Table S2).

Overall, these results show that the buckypaper strategy can partially mitigate the usual trade‐off between electrode thickness and active‐site utilization in TEMPO‐based cathodes. Further improvements in through‐thickness electron/ion transport will, however, be required to translate this proof of concept into areal capacities approaching those of fully practical Li‐ion cells.

## 3 Conclusion

In summary, we demonstrate that post‐synthetic click‐functionalization of imine‐linked COFs with TEMPO radicals provides an effective strategy to access high‐voltage porous organic cathodes with accessible and reversibly addressable redox sites. The resulting **TEMPO‐TB** and **TEMPO‐TP** frameworks retain their crystalline and porous structure after functionalization, despite a reduction in surface area, and exhibit stable p‐type redox activity at ∼3.6–3.7 V vs. Li/Li^+^, confirming the potential of radical‐functionalized COFs as high‐voltage cathode materials. **TEMPO‐TB** and **TEMPO‐TP** show no significant differences in electrochemical performance, indicating that the triazine units are electrochemically inactive within the investigated potential window. More importantly, our results reveal that the performance of TEMPO‐based porous cathodes is governed not only by framework design but also by the coupled interaction between the radical‐bearing host and the electrolyte anion. Among the electrolytes investigated, LiDFOB provides the most favorable overall performance, delivering the best compromise between specific capacity, rate capability, and cycling stability. This behavior arises from a combined effect of anion transport, interfacial charge‐transfer kinetics, redox‐site accessibility, and pseudocapacitive contributions. Notably, the buckypaper electrode strategy enables high active‐material fractions of up to 80 wt.% and mass loadings of 40 mg cm^−2^ while maintaining meaningful gravimetric performance and achieving an areal capacity of 1.3 mAh cm^−2^, the highest reported for this family of materials. These findings establish electrolyte–framework matching as a key design principle for porous radical electrodes and advance the development of high‐voltage TEMPO‐based COF cathode materials.

## Conflicts of Interest

The authors declare no conflicts of interest.

## Supporting information




**Supporting File**: advs76112‐sup‐0001‐SuppMat.pdf.

## Data Availability

The data that support the findings of this study are available in the Supporting Information of this article.
