# Supplementary material for: Electrolyte–Framework Matching in High‐Voltage TEMPO‐COF Cathodes for Lithium Batteries
Source: Adv Sci (Weinh). 2026 Jun 18:e76112. Online ahead of print. doi: 10.1002/advs.76112 (PMC13336897; doi:10.1002/advs.76112)
Supplement: Supplementary file 1 — Supporting File: advs76112‐sup‐0001‐SuppMat.pdf. [file ADVS-9999-e76112-s001.pdf]

# Supporting Information

## Electrolyte–Framework Matching in High-Voltage TEMPO-COF Cathodes for Lithium Batteries

Marilyn Esclance DMello,[a*,†] Nagaraj Patil,[b,†] Fanni Fekecs,[a] Sergio Pinilla,[b] Rebeca Marcilla,[b*] and Manuel Souto [a,c*]

Marilyn Esclance DMello, Fanni Fekecs and Manuel Souto

CiQUS, Centro Singular de Investigacion en Química Biolóxica e Materiais Moleculares,

Departamento de Química-Fısica, Universidade de Santiago de Compostela,

15705 Santiago de Compostela, Spain.

E-mail: manuel.souto.salom@usc.es, marilynesclance.dmello@usc.es

Nagaraj Patil, Sergio Pinilla and Rebeca Marcilla.

Electrochemical Processes Unit, IMDEA Energy, Avda. Ramón de La Sagra 3,

28935 Móstoles, Spain.

E-mail: rebeca.marcilla@imdea.org

Manuel Souto

Oportunius, Galician Innovation Agency (GAIN), 15702 Santiago de Compostela, Spain

**Table of contents**


| Sr. No. | Contents | Pg. No. |
|---|---|---|
| **1.** | General procedures and experimental details | S2 |
| **2.** | Synthesis of TEMPO-COFs | S3 |
| **3.** | Physicochemical characterization of TEMPO-COFs | S6 |
| **4.** | Calculation of spins per molecule from EPR | S10 |
| **5.** | Theoretical capacity calculation based on spins per molecule from EPR | S10 |
| **6.** | Electrochemical studies | S12 |
| **7.** | References | S41 |

## 1. General procedures and experimental details

### 1.1. Chemicals and reagents

1,3,5-tris(4-aminophenyl)benzene (TB), 2,4,6-tris(4-aminophenyl)-1,3,5-triazine (TP) and 2,5-bis(prop-2-ynoxy)terephthalaldehyde (BPTA) were purchased from BLD Pharma. 4-Azido-2,2,6,6-tetramethylpiperidine 1-oxyl radical ($N_3$-TEMPO) was purchased from ABCR Germany. 1,2-dichlorobenzene and *n*-butanol were received from TCI-Chemicals, Europe. Tetrahydrofuran, triethylamine and acetonitrile were purchased from Fischer Scientific. Lithium foil (600 μm), and Celgard (2500) were received from Sigma-Aldrich. Nanografi was chosen to purchase reduced graphene oxide (RGO) and single-walled carbon nanotubes (SWCNTs). Battery-grade lithium salts ($LiPF_6$, $LiClO_4$, $LiBF_4$, LiDFOB, and LiTFSI) and the solvents ethylene carbonate (EC) and dimethyl carbonate (DMC) were purchased from commercial suppliers, Solvionic, Sigma-Aldrich; the salts were used in dried form, and the solvents were pre-dried over molecular sieves before preparing the respective electrolyte solutions.

### 1.2. Characterization

Powder X-ray diffraction (PXRD) patterns were recorded using a Rigaku MiniFlex 600-C X-ray diffractometer using Cu Kα radiation (λ = 1.54056 Å). The X-ray tube was operated at a voltage of 40 kV and a current of 15 mA. Data were collected in the ca. $3^o \leq 2\theta \leq 40^o$ range was used to identify the crystalline phases of the materials. Fourier transform infrared (FTIR) spectra were recorded using powdered samples in Bruker Tensor 27 spectrometer coupled with a Specac Golden Gate Diamond attenuated total reflectance (ATR) accessory in the range of 4000 and 350 $cm^{-1}$. Micromeritics 3Flex apparatus was used to measure the specific BET surface area by obtaining $N_2$ adsorption isotherms at 77 K and by using the Brunauer-Emmett-Teller (BET) method and pore size distributions were obtained using the non-local density functional theory (NLDFT) method. The samples were activated overnight at 100 °C and $10^{-6}$ Torr prior to the analysis. Field emission scanning electron microscopy (FE-SEM) with a microscopy JEOL JSM-7800F Prime and with an accelerating voltage of 15 kV was used to study the morphology of the materials. Thermogravimetric analysis (TGA) was carried out with a Shimadzu TGA 50 equipment in the 25–700 ºC temperature range under a 5 ºC $min^{-1}$ scan rate and an $N_2$ flow of 20 $mL \cdot min^{-1}$. EPR measurements were performed in a Bruker ESP-300E spectrometer operating in the X band 9.861 GHz and with a field modulation of 2 GHz at room temperature.

## 2. Synthesis of TEMPO-COFs

### 2.1. Synthesis of TB COF

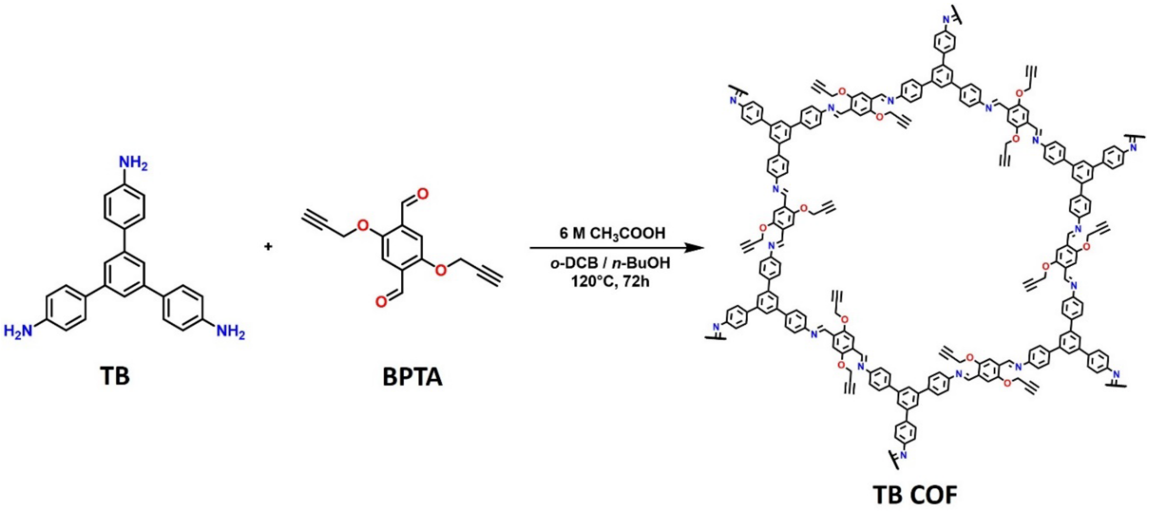


A Schlenk-tube was charged with TB (176 mg, 0.5 mmol) and BPTA (182 mg, 0.75 mmol), which were dissolved in a mixture of 1,2-dichlorobenzene and *n*-butanol (1:1, 12.4 mL). Then, 6 M aqueous acetic acid (0.6 mL) was added as a catalyst. The resulting suspension was sonicated for 10 minutes at room temperature. The reaction mixture was subjected to three freeze-pump-thaw cycles, backfilled with Ar, and sealed. The reaction mixture was then heated at 120 °C for 72 h in an oven. After cooling to room temperature, the solid was collected by filtration and washed with THF. The product was then dried at 120 °C under vacuum for 24 h to afford a brown solid (**TB COF**, 318 mg, 93% yield).

### 2.2. Synthesis of TP COF

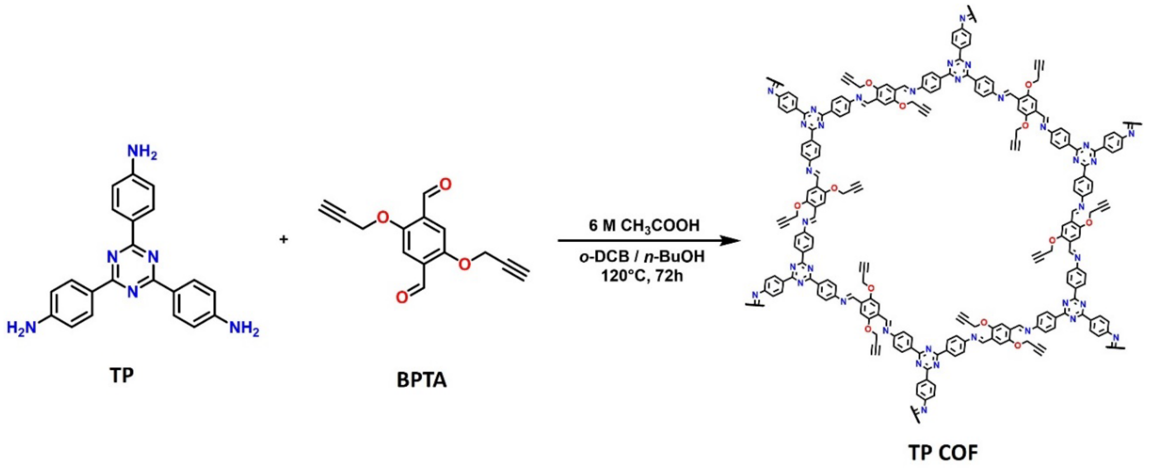


A Schlenk-tube was charged with TP (708 mg, 2.0 mmol) and BPTA (726 mg, 3.0 mmol), which were dissolved in a mixture of 1,2-dichlorobenzene and n-butanol (1:1, 50 mL). Then, 6 M aqueous acetic acid (2.5 mL) was added as a catalyst. The resulting suspension was

sonicated for 10 minutes at room temperature. The reaction mixture was subjected to three freeze-pump-thaw cycles, backfilled with Ar, and sealed. The reaction mixture was then heated at 120 °C for 72 h in an oven. After cooling to room temperature, the solid was collected by filtration and washed with THF. The product was then dried at 120 °C under vacuum for 24 h to afford a brown solid (**TP COF**, 1.03 g, 75% yield).

### 2.3. Synthesis of TEMPO-TB COF

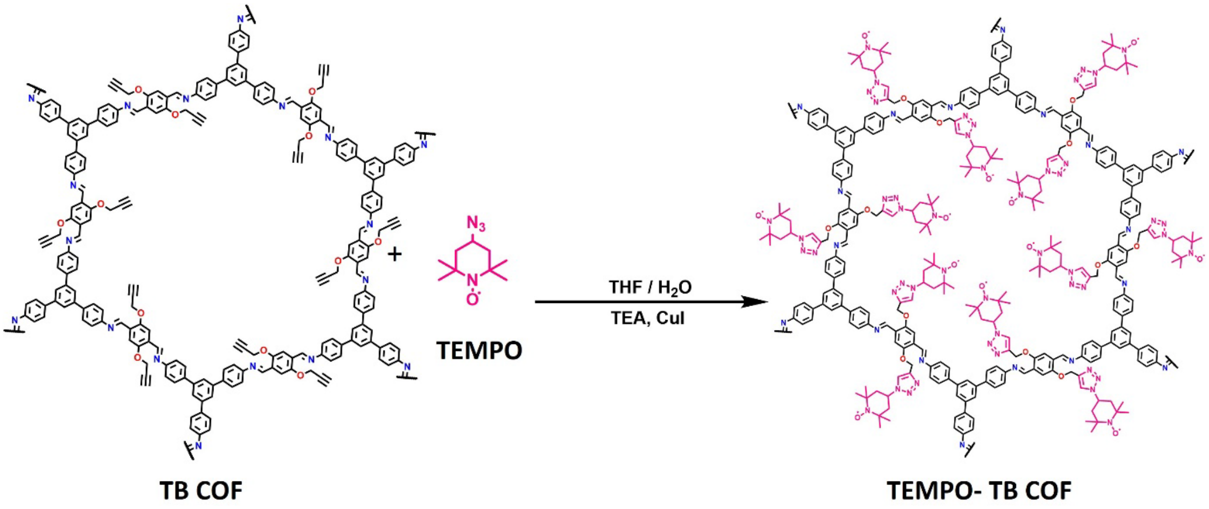


**TB COF** (137 mg, 0.2 mmol) was added to a Schlenk-tube, and it was suspended in a mixture of THF (12.3 mL) and $H_2O$ (2.05 mL). Subsequently, $N_3$-TEMPO (205 mg, 1.04 mmol), triethylamine (TEA) (51.3 µL, 0.368 mmol), and CuI (5.14 mg, 0.027 mmol) were added. The reaction mixture was subjected to three freeze-pump-thaw cycles, backfilled with Ar and then sealed. The reaction mixture was then stirred at room temperature for 120 h. The resulting precipitate was filtered, washed with THF and acetonitrile, and dried under vacuum for 24 h to afford an olive-brown solid (**TEMPO-TB COF**, 193 mg, 75% yield).

## 2.4. Synthesis of TEMPO-TP COF

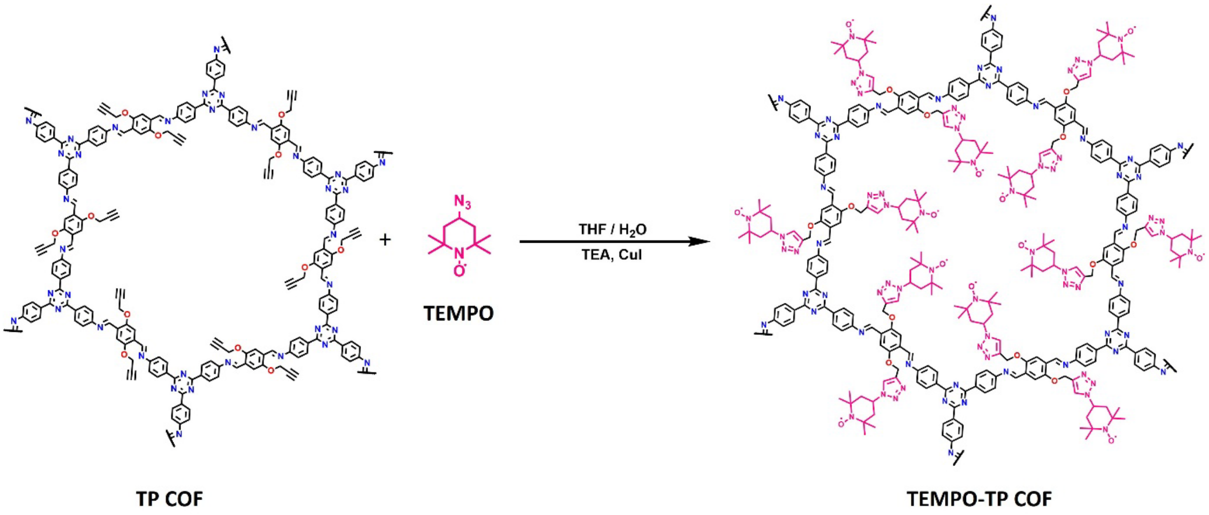


TP COF (62.1 mg, 0.09 mmol) was added to a Schlenk-tube, and it was suspended in a mixture of THF (5.54 mL) and $H_2O$ (0.92 mL). Subsequently, $N_3$-TEMPO (92.3 mg, 0.468 mmol), triethylamine (TEA) (16.8 µL, 0.166 mmol), and CuI (2.31 mg, 0.012 mmol) were added. The reaction mixture was subjected to three freeze-pump-thaw cycles, backfilled with Ar and then sealed. The reaction mixture was then stirred at room temperature for 120 h. The resulting precipitate was filtered, washed with THF and acetonitrile, and dried under vacuum for 24 h to afford an olive-brown solid (**TEMPO-TP COF**, 84 mg, 73% yield).

## 3. Physicochemical characterization of TEMPO-COFs

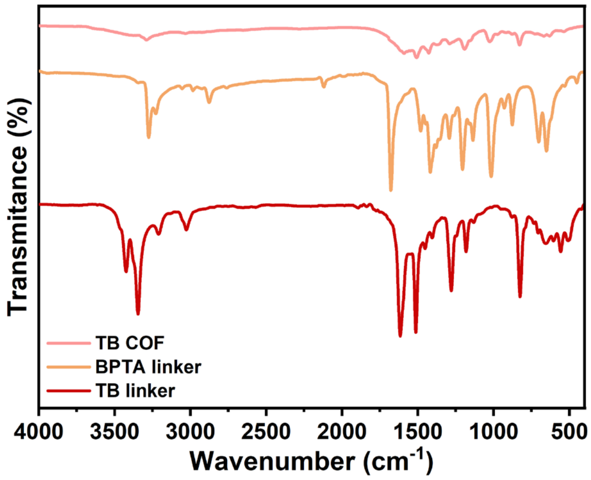


**Figure S1**. FT-IR spectra of TB, BPTA, and **TB** COF.

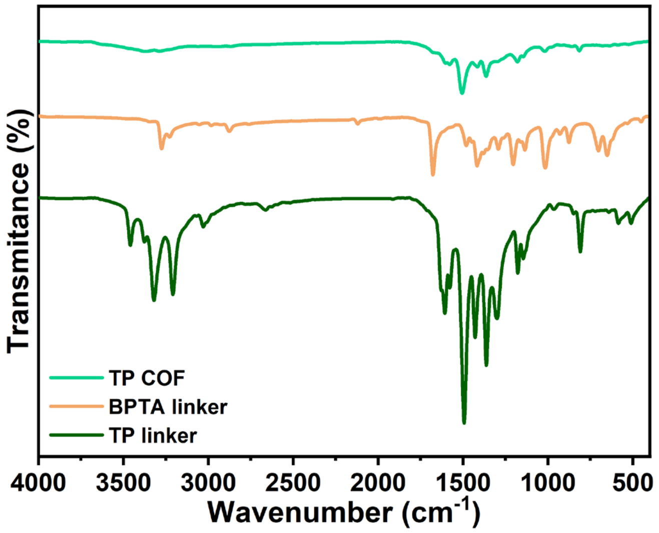


**Figure S2**. FT-IR spectra of TP, BPTA, and **TP** COF.

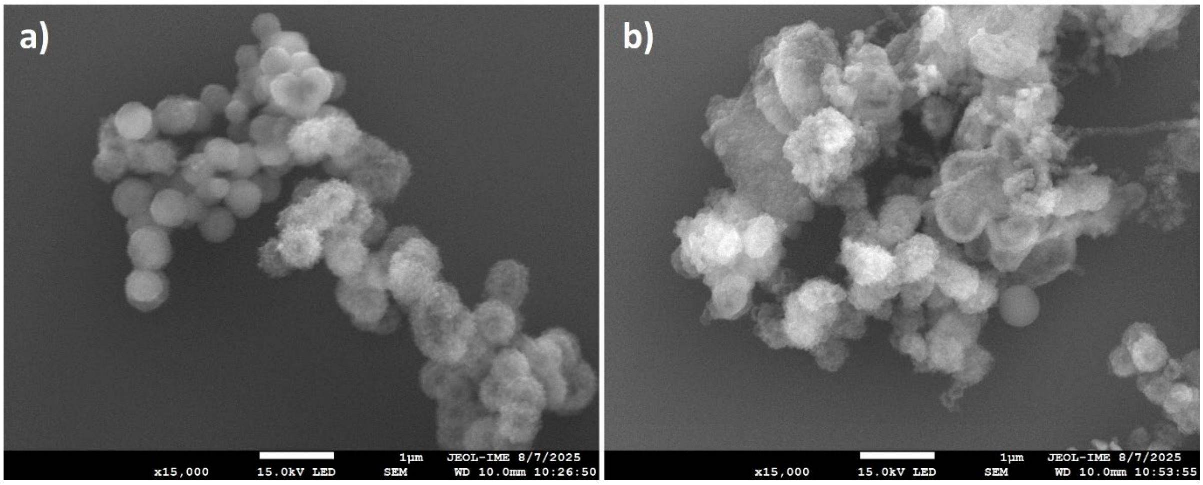


**Figure S3**. SEM images of a) **TB** COF and b) **TEMPO-TB** COF.

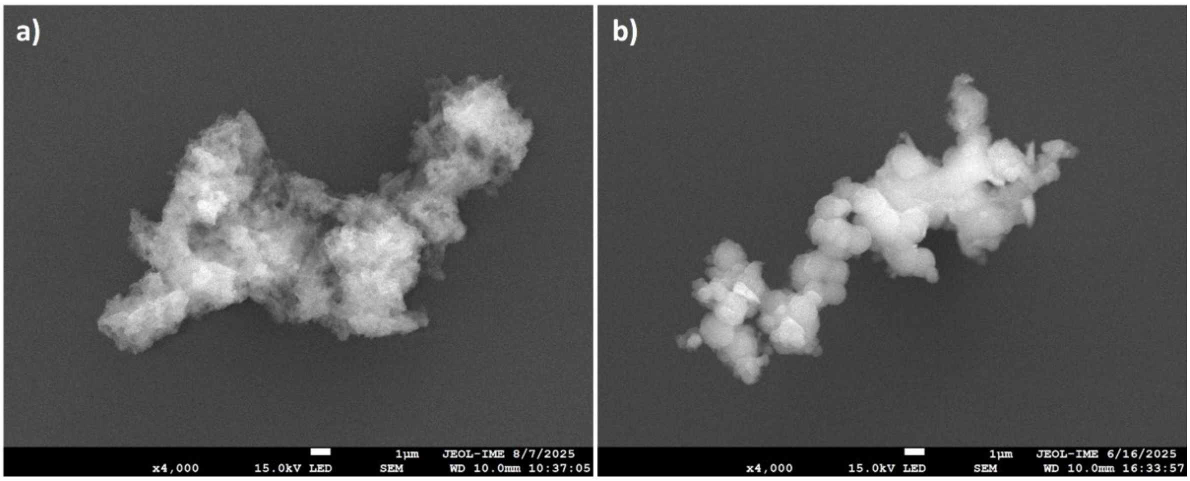


**Figure S4**. SEM images of a) **TP** COF and b) **TEMPO-TP** COF.

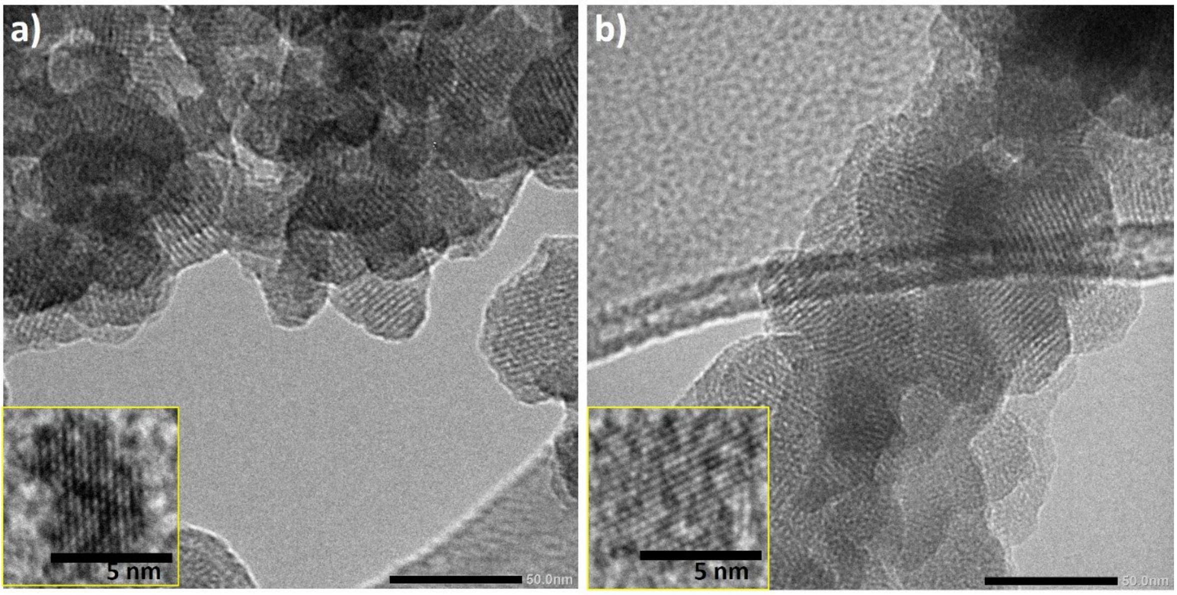


**Figure S5.** HRTEM images of a) **TEMPO-TB** COF and b) **TEMPO-TP** COF.

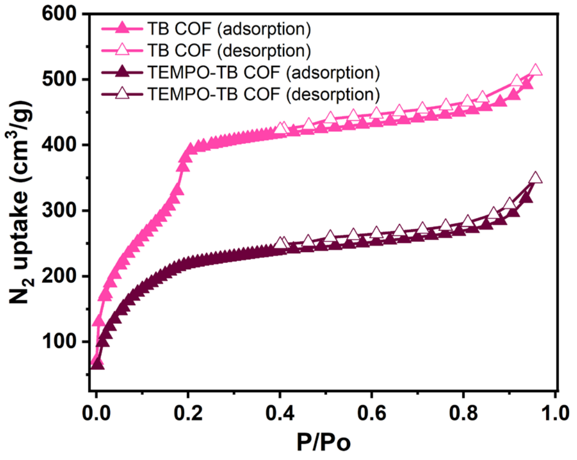


**Figure S6**. $N_2$ sorption isotherms of **TB** COF and **TEMPO-TB** COF at 77 K.

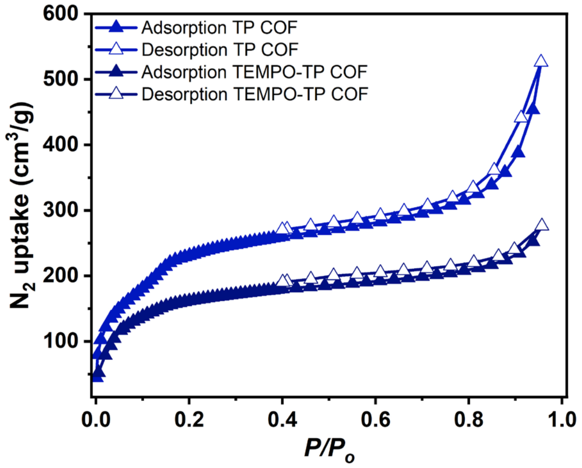


**Figure S7**. $N_2$ sorption isotherms of **TP** COF and **TEMPO-TP** COF at 77 K.

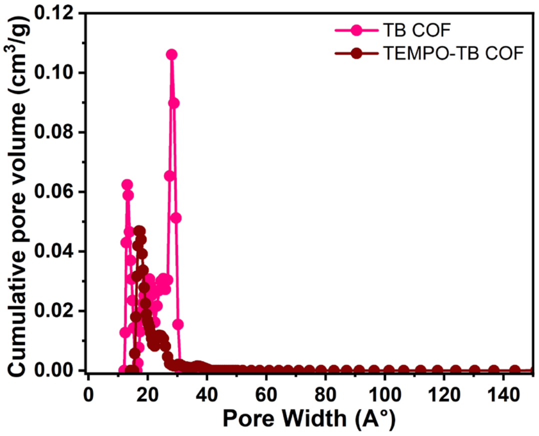


**Figure S8**. Pore size distribution of **TB** COF and **TEMPO-TB** COF.

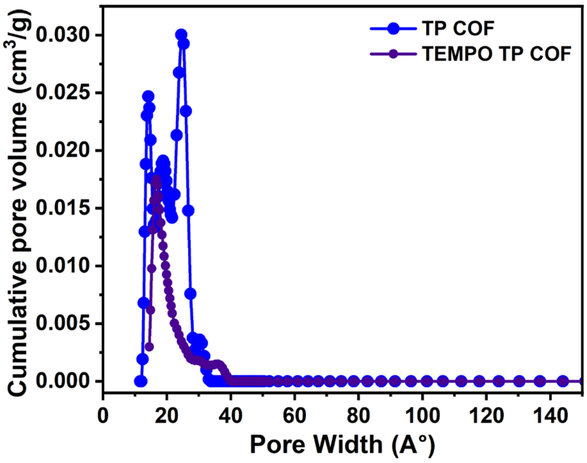


**Figure S9**. Pore size distribution of **TP** COF and **TEMPO-TP** COF.

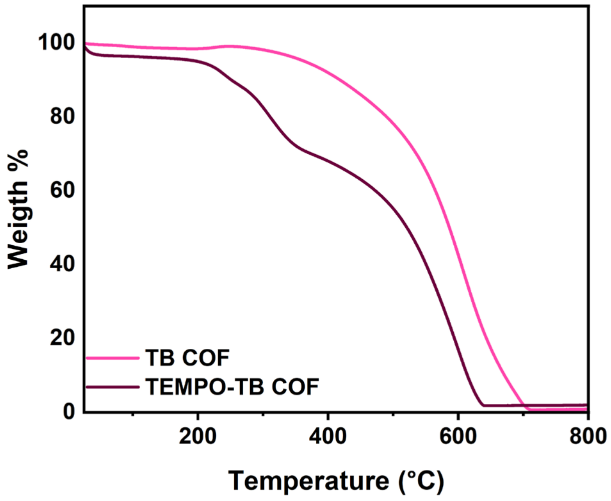


**Figure S10**. TGA of **TB** COF and **TEMPO-TB** COF.

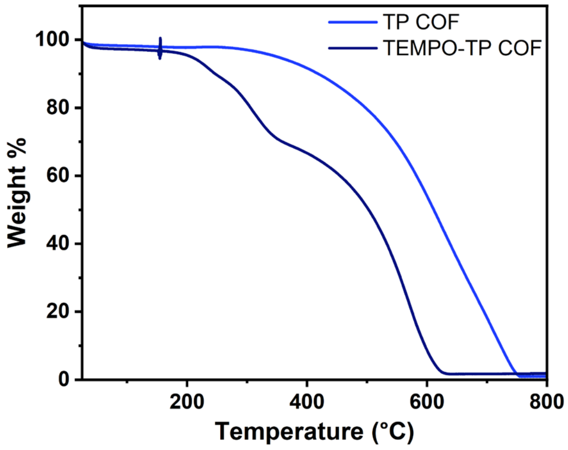


**Figure S11**. TGA of **TP** COF and **TEMPO-TP** COF.

## 4. Calculation of spins per molecule from EPR

**TEMPO-TP COF:**

The number of spins per molecule was calculated by comparison with standard solutions of 1,1-diphenyl 2-picrylhydrazyl (DPPH) in toluene. Fresh solutions with precisely known concentrations were measured before and after every COF sample, under identical conditions. Then, the area of the EPR signal of the COF and the standard were compared to obtain the absolute number of spins in the sample.

- Spins/mol = spins/g × g/mol = $(1 \times 10^{21}) \times 2573.04 = 2.57304 \times 10^{24}$ spins/mol
- Spins/molecule = (spins/mol) /$N_A$= $(2.57304 \times 10^{24}) / 6.022 \times 10^{23}$
- For **TEMPO-TB**, the calculated **spins/unit cell = 4.2**

Similarly, in the case of **TEMPO-TB** COF, the calculated **spins/unit cell = 4.5**

## 5. Theoretical capacity calculation based on spins per molecule from EPR.

- Formula to calculate the theoretical capacity: (mAh/g) = (n × F) / (3.6 × M).
- Molecular weights (M) per unit cell:

**TP** COF = 1405.51 g/mol

**TEMPO-TP** COF = 2573.04 g/mol

**TB** COF = 1321.47 g/mol

**TEMPO-TB** COF = 2489 g/mol

- Active sites (n) (from the nitroxyl radicals (TEMPO), each transferring 1 electron):

**TEMPO-TP** COF = 4.2

**TEMPO-TB** COF = 4.5

- Faraday's constant (F) = 96485 C/mol.
- Theoretical capacities:
  **TEMPO-TP**= 43.7 mA h/g
  **TEMPO-TB**= 48.5 mA h/g.

## 6. Electrochemical methodology and characterization

### 6.1. Electrode preparation

**TEMPO-COFs low mass loading buckypaper preparation**: The process of preparing the buckypapers involved several steps. The electrode composition was 60:30:10 of COF, single-walled carbon nanotubes (SWCNTs) and reduced graphene oxide (rGO), respectively. First, the SWCNTs were weighed and dispersed in isopropanol, followed by 15 minutes of tip sonication to ensure even distribution. Next, rGO and COF were weighed and ground in a mortar and pestle and then added to the SWCNTs dispersion in isopropanol. This mixture was left to stir overnight at room temperature. Following stirring, an additional hour of bath sonication was carried out, followed by vacuum filtration using a hydrophobic Whatman filter paper (45 mm). The buckypaper was first left to dry under vacuum at room temperature for at least 30 minutes, separated from the filter paper and dried overnight at 80 °C under vacuum. The electrodes were fabricated with a mass loading of active-material (TEMPO-COFs) of ~2 mg $cm^{-2}$. As a commonly used procedure for organic batteries, specific capacities were calculated based on the amount of the active material. In this case, 8 mm circular discs were cut and used to assemble the coin cells.

**TEMPO-COFs high mass loading buckypaper preparation**: In this case, the electrodes were fabricated with a mass loading of active-material (TEMPO-COFs) of 5 mg $cm^{-2}$, 10 mg $cm^{-2}$, 20 mg $cm^{-2}$ and 40 mg $cm^{-2}$. The composition of the cathodes was 80:15:5 (active material:SWCNT:rGO). First, the SWCNTs were weighed and dispersed in isopropanol, followed by 15 minutes of tip sonication to ensure even distribution. Next, rGO and COF were weighed and ground in a mortar and pestle and then added to the SWCNTs dispersion in isopropanol. This mixture was left to stir overnight at room temperature. Following stirring, an additional hour of bath sonication was carried out, followed by vacuum filtration using a hydrophobic Whatman filter paper (15 mm). The buckypaper was first left to dry under vacuum at room temperature for at least 45 minutes, separated from the filter paper and dried overnight at 80 °C under vacuum. All the high mass loading buckypaper electrodes were compressed using a pellet/film compression machine. Following this, 6 mm circular discs were cut and used to assemble the coin cells.

### 6.2. Li-ion half-cell fabrication

The prepared buckypaper electrodes (TEMPO-COF, pristine COF with low mass loading (2 mg/cm$^2$) and low active mass content (60:30:10) were cut into circular discs with 8 mm of diameter, weighed, and moved to an Ar-filled glovebox to be used in the cell assembly. Whereas, in the case of buckypapers of high mass loading (5-40 mg/cm$^2$) and high active mass content (80:15:5), they were cut into circular discs of 6 mm diameter. Half-cells were assembled using a CR2032 coin-cell set-up with the self-standing buckypaper COF as the positive electrode. Lithium metal foil, with a 10 mm diameter, was used as the reference and counter electrode with a Whatman (GF/A) glass fibre separator soaked with the liquid electrolyte (150 $\mu$L), 1 M LiX (X= $PF_6$, $ClO_4$, $BF_4$, DFOB, TFSI) in a mixture of ethylene carbonate (EC) and dimethyl carbonate (DMC), EC/DMC (3/7 v/v).

In order to assess the n-type activity of the TEMPO functionalities, a glyme-based electrolyte (1 M LiTFSI in TEGDME) was used for electrochemical evaluation. Notably, such electrolytes are widely reported to better stabilize reduced species and low-potential chemistries.

### 6.3. Electrochemical measurements

**Cyclic voltammetry.** The electrochemical behavior of TEMPO-COF buckypaper electrodes (60:30:10 wt.% COF:SWCNTs:rGO) was investigated by cyclic voltammetry (CV) in coin-type lithium cells using a Bio-Logic potentiostat/galvanostat. Initial CV measurements were recorded at a scan rate of 0.1 mV s$^{-1}$, while additional measurements at scan rates from 0.1 to 1 mV s$^{-1}$ were performed on the same electrodes to assess the electrode kinetics.

*a) Power-law Analysis:*[1] CV data generally follow a power-law dependence between the peak current ($i_p$) and scan rate ($v$). This analysis allows the determination of the $b$-value from the slope of a plot of log ($i_p$)versus log ($v$), providing insight into the kinetics of the redox process. The relationship is described by:

$$i_p = av^b \quad \text{(Equation S1)}$$

and

$$log i_p = \log a + b\log v \quad \text{(Equation S2)}$$

where $a$ and $b$ are adjustable parameters. The $b$-value reflects the dominant charge-storage mechanism. A value of $b = 1$ indicates a surface-controlled process, typically associated with fast surface redox reactions and electric double-layer capacitance (EDLC), whereas $b = 0.5$ is

characteristic of diffusion-limited faradaic processes occurring within the bulk of the electrode, as commonly observed in battery-type systems.

*b) Dunn´s method to evaluate capacity contributions:*[2] Dunn's method was used to quantitatively distinguish the capacitive (surface) and diffusion-controlled contributions to the current response at different scan rates. At a given potential ($V$), the current $i$ can be expressed as the sum of a capacitive term and a diffusion-controlled term according to:

$$i(V) = k_1 v + k_2 v^{\frac{1}{2}} \quad \text{(Equation S3)}$$

$$\frac{i(V)}{v^{1/2}} = k_1 v^{1/2} + k_2 \quad \text{(Equation S4)}$$

where $k_1$ and $k_2$ are constants. From the linear relationship between $i(V)/v^{1/2}$and $v^{1/2}$, the values of $k_1$ and $k_2$ can be obtained at each potential. These parameters were then used to quantify and plot the capacitive (surface) and diffusion-controlled contributions at different scan rates.

**Electrochemical impedance spectroscopy (EIS).** The resistive and capacitive properties of the Li//TEMPO COFs half-cells were investigated by electrochemical impedance spectroscopy (EIS). Measurements were performed over a frequency range of 0.01 Hz to 1 × $10^6$ Hz using a sinusoidal perturbation with an amplitude of 10 mV ($V_{rms}$ ≈ 7.07 mV) at selected potentials during both charge and discharge at 25 °C.

EIS spectra were modeled with an equivalent circuit comprising the uncompensated series resistance ($R_{series}$), followed by a parallel interfacial network between $\mathrm{CPE_1}$ and a branch containing the charge-transfer resistance ($R_{CT}$) in series with the Warburg diffusion element ($W_1$), and finally a second constant phase element ($\mathrm{CPE_2}$) connected in series.

**Galvanostatic intermittent titration technique (GITT).** Galvanostatic intermittent titration technique (GITT) measurements were performed at 25 °C to estimate the solid-state chemical diffusivity of anions ($D_{anion^-}$) within the COF buckypaper electrodes. Before the GITT experiment, Li//COF cells were pre-cycled and brought to the fully discharged state. The cells were then charged at C/5 for 600 s, followed by a 120 min relaxation period. This pulse–rest sequence was repeated until the cell voltage reached 3.95 V. The same protocol was subsequently applied during discharge down to 3.0 V. The anion diffusivity was calculated according to:

$$D_{anion} = \frac{4L^2}{\tau\pi}(\Delta E_s/\Delta E_t)^2 \qquad \text{(Equation S5)}$$

where $L$(cm) is the anion diffusion length, approximated by the electrode thickness (100 and 110 $\mu$m for TEMPO-TP and TEMPO-TB COFs, respectively), $\tau$ is the duration of the current pulse (s), and $\Delta E_s$ and $\Delta E_t$ are the steady-state voltage change and the voltage change during the current pulse (excluding the IR drop), respectively.

**Charge–discharge experiments.** Cycling stability at 1C and rate capability from C/5 to 60C were evaluated by galvanostatic charge–discharge measurements in coin-type Li cells using a Neware battery cycler at 25 °C.

**Performance metrics for Li-cells.** For polymer-based organic batteries, the gravimetric specific capacity and applied current rate (C-rate) are generally normalized to the mass of the polymeric active material in the working electrode, here corresponding to the COF mass, unless stated otherwise. Areal performance values were calculated by multiplying the gravimetric values by the corresponding areal mass loading.

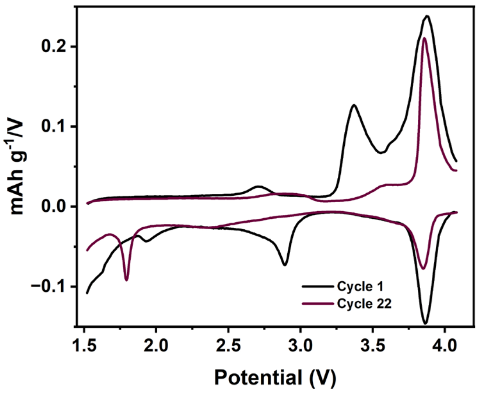


**Figure S12.** Differential capacity (dQ/dV) versus voltage profiles of a Li||TEMPO-TB COF half-cell cycled in 1 M LiTFSI/TEGDME at a 1C rate, showing the 1$^{st}$ and 22$^{nd}$ cycles. Electrode active mass loading ~2 mg cm$^{-2}$. Electrode composition 60:30:10 wt.%.

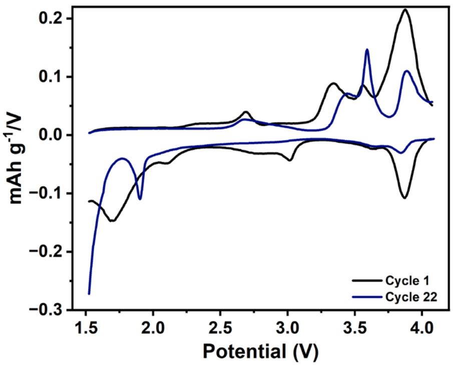


**Figure S13.** Differential capacity (dQ/dV) versus voltage profiles of a Li||TEMPO-TP COF half-cell cycled in 1 M LiTFSI/TEGDME at a 1C rate, showing the 1st and 22nd cycles. Electrode active mass loading ~2 mg cm⁻². Electrode composition 60:30:10 wt.%.

__Note:__ Glyme-based systems are generally more suitable for probing reduction processes, as they provide enhanced stability toward highly reduced and reactive intermediates. As shown in Figures S12 and S13, a reduction peak centered at ~3.1 V vs. $Li/Li^+$, attributable to the $TEMPO^{\bullet}/TEMPO^-$ process, is observed during the initial scan together with the corresponding oxidation feature. However, this redox couple rapidly diminishes upon cycling and is no longer visible after ~22 cycles, in agreement with previous reports and reflecting the limited stability and poor reversibility of the reduced TEMPO state.

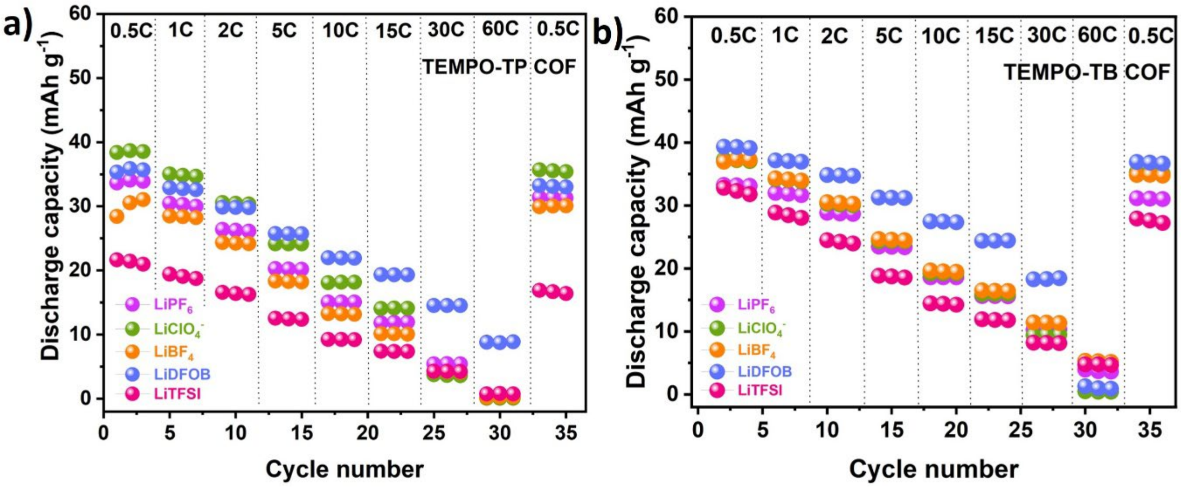


**Figure S14.** Discharge capacity vs cycle number at different C-rates of a) **TEMPO-TP** COF and b) **TEMPO-TB** COF. Electrode active mass loading ~2 mg cm⁻². Electrode composition 60:30:10 wt.%.

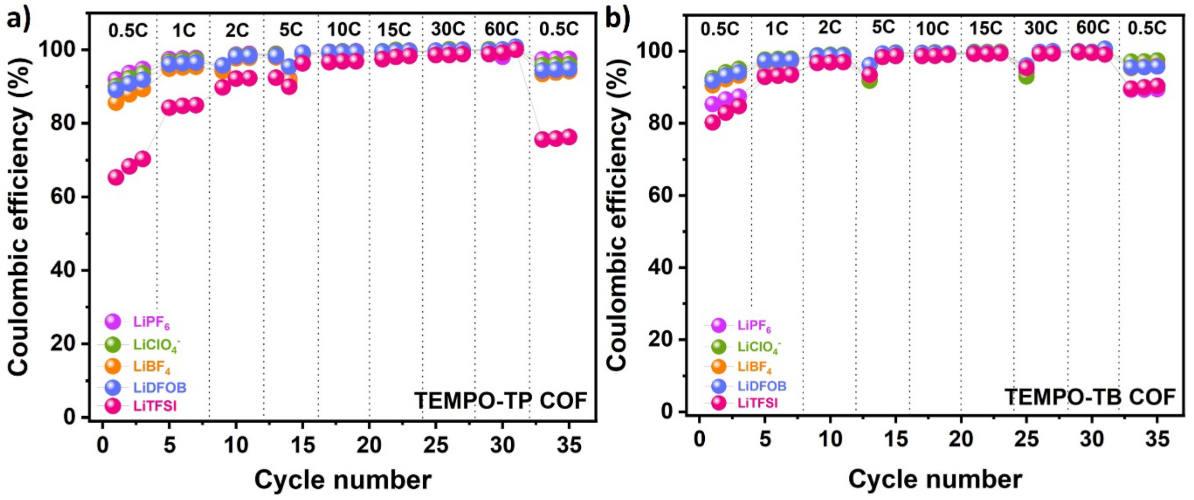


**Figure S15.** Coulombic efficiency vs cycle number at different C-rates of a) **TEMPO-TP** COF and b) **TEMPO-TB** COF. Electrode active mass loading ~2 mg cm$^{-2}$. Electrode composition 60:30:10 wt.%.

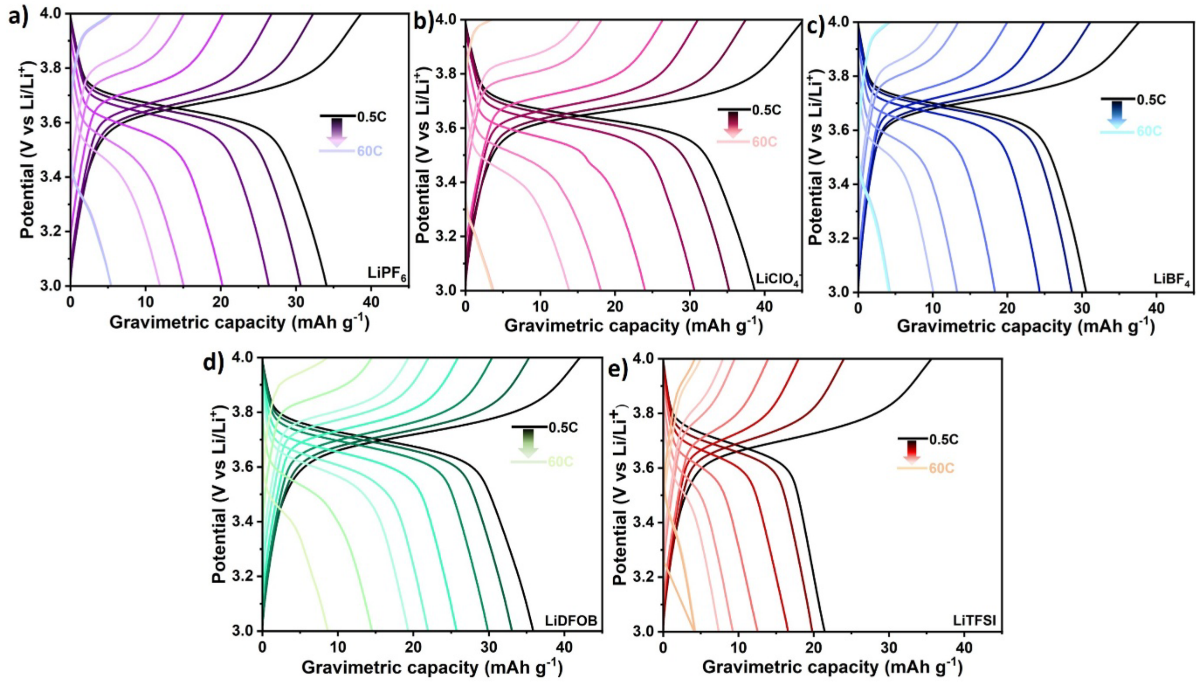


**Figure S16.** GCPL profiles of the **TEMPO-TP** COF electrodes at different C-rates in a) $LiPF_6$, b) $LiClO_4$, c) $LiBF_4$, d) LiDFOB and e) LiTFSI electrolytes. Electrode active mass loading ~2 mg cm$^{-2}$. Electrode composition 60:30:10 wt.%.

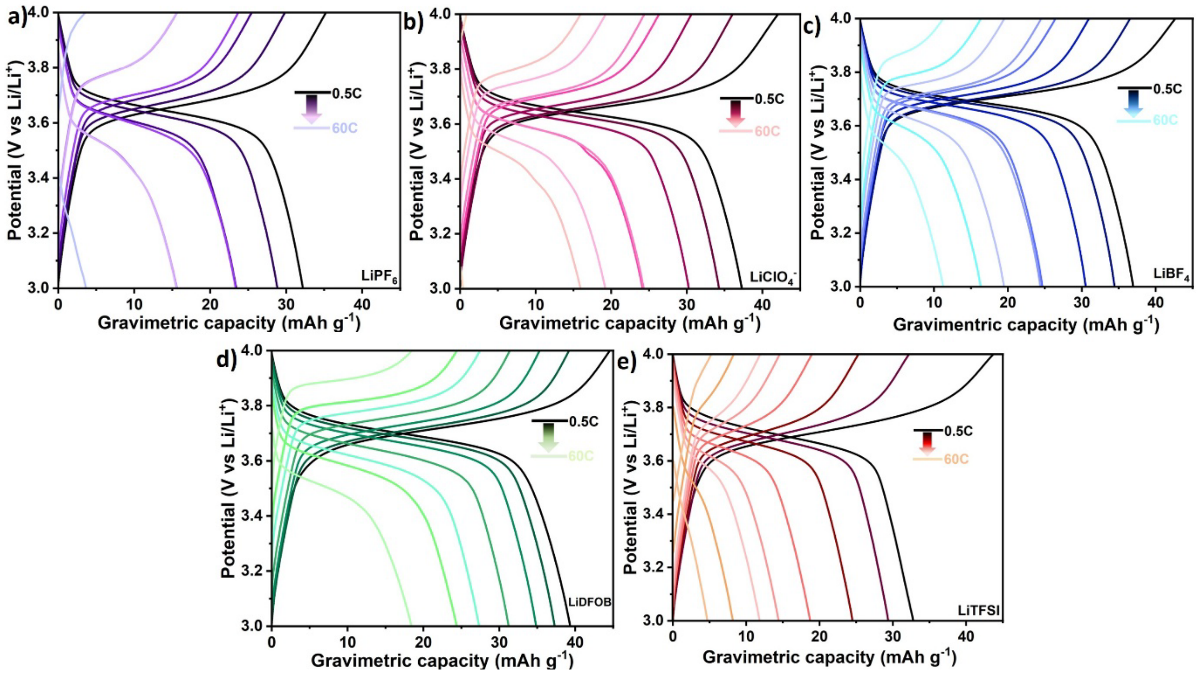


**Figure S17.** GCPL profiles of the **TEMPO-TB** COF electrodes at different C-rates in a) $LiPF_6$, b) $LiClO_4$, c) $LiBF_4$, d) LiDFOB and e) LiTFSI electrolytes. Electrode active mass loading ~2 mg cm$^{-2}$. Electrode composition 60:30:10 wt.%.

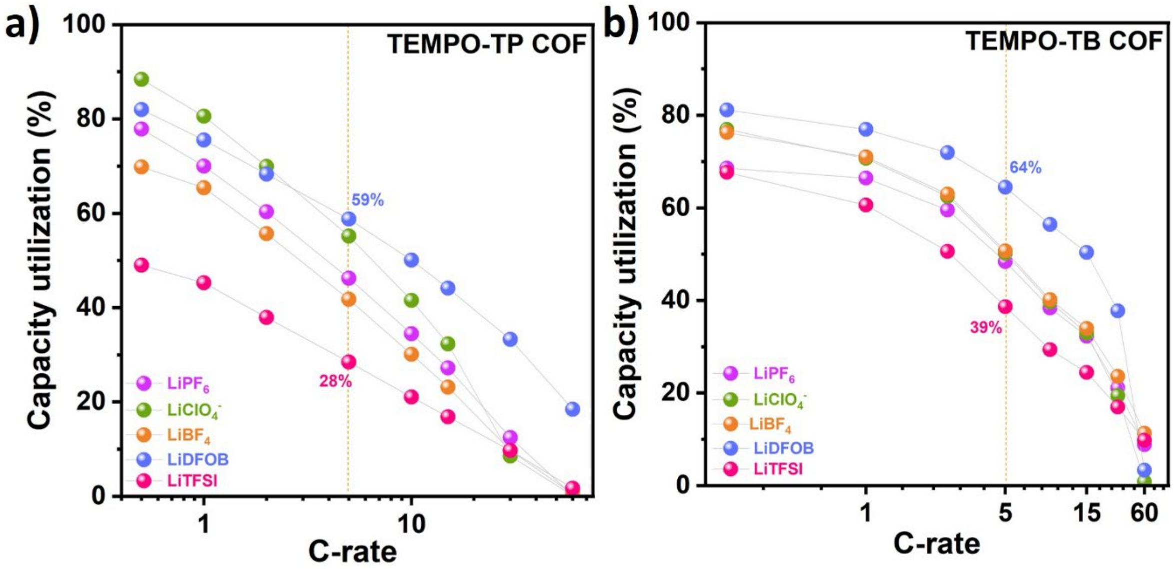


**Figure S18.** Capacity utilization of a) **TEMPO-TP** COF and b) **TEMPO-TB** COF buckypaper electrodes.

### 6.4. Kinetic analyses:

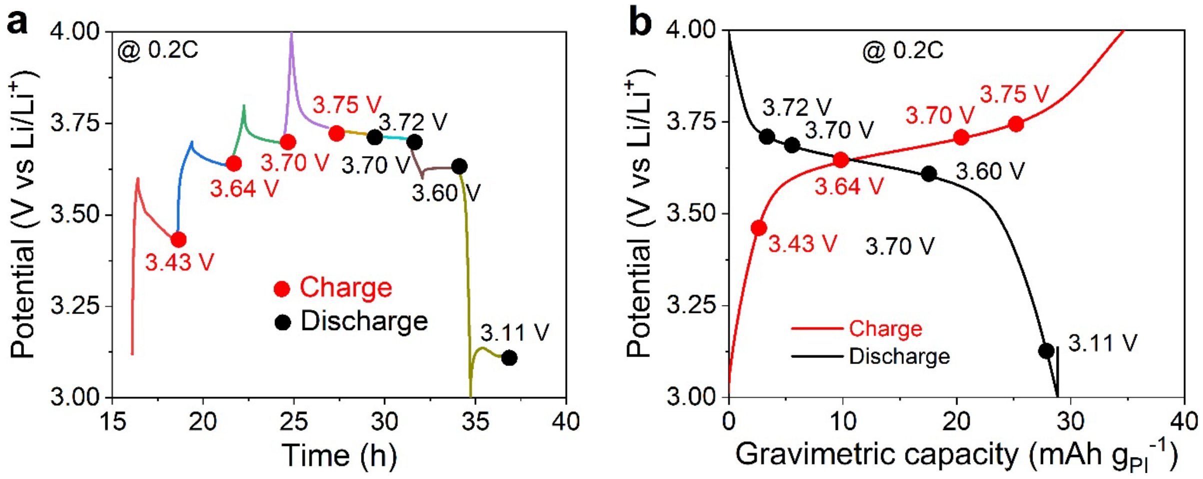


**Figure S19.** Charging and discharging **TEMPO-TP** COF buckypaper electrode in Li half-cell at 0.2C to different potentials for the EIS measurements. a) potential vs time, and b) potential vs capacity profiles. Electrode active mass loading ~2 mg cm$^{-2}$. Mass loading ~2 mg cm$^{-2}$. Composition 60:30:10 wt.%.

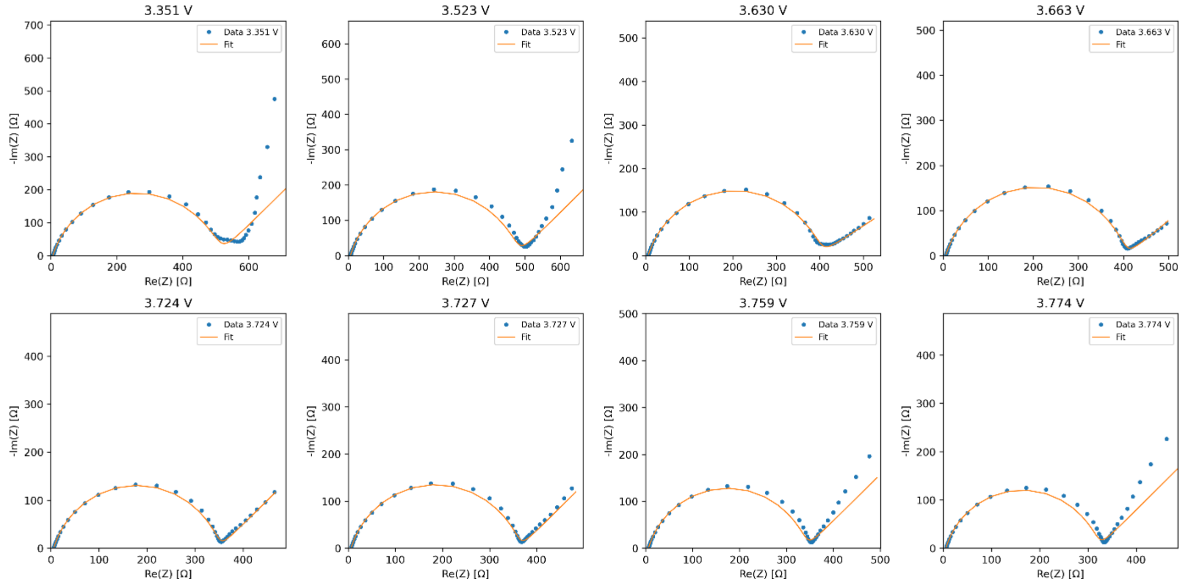


**Figure S20.** Nyquist plots at different potentials during the charging and discharging steps for **TEMPO-TP** COF buckypaper electrode in Li half-cell in **PF$_6$-based** electrolyte. Mass loading ~2 mg cm$^{-2}$. Composition 60:30:10 wt.%.

<u>**Note:**</u> In LiPF$_6$, $R_s$ remains nearly constant at ~10–11 Ω, whereas $R_{ct}$ varies strongly with potential, decreasing from ~580 Ω at 3.43 V to ~220–280 Ω across the main charging plateau (3.64–3.75 V), remaining relatively low near the corresponding discharge plateau (3.72–3.70 V), and increasing sharply again at deep discharge to ~800 Ω at 3.11 V.

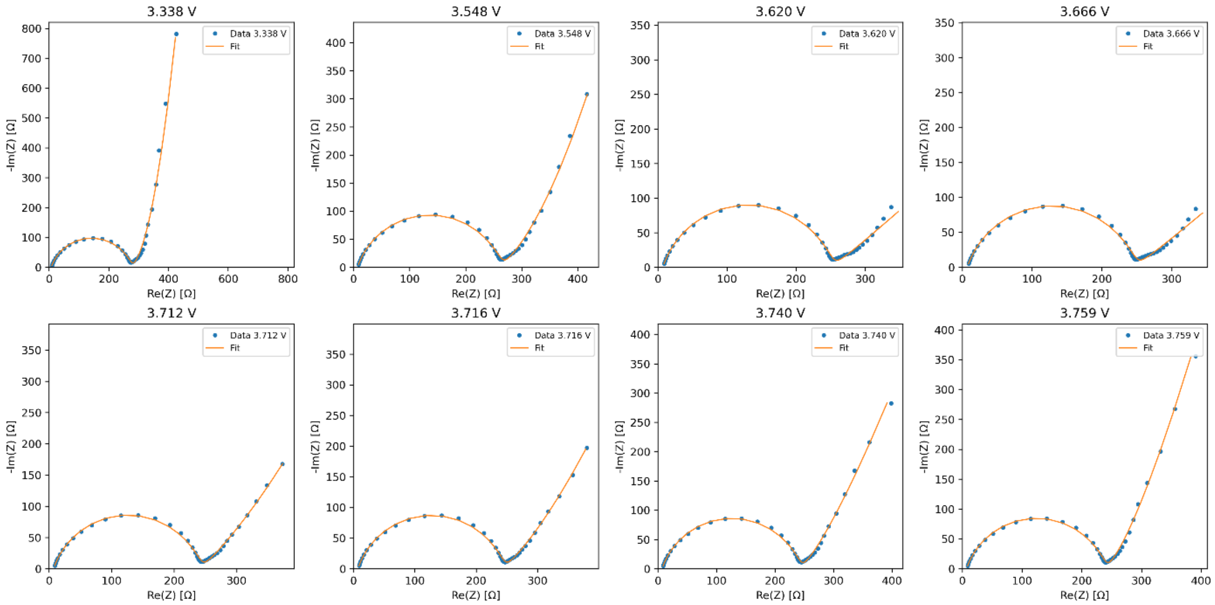


**Figure S21.** Nyquist plots at different potentials during the charging and discharging steps for **TEMPO-TP** COF buckypaper electrode in Li half-cell in **$ClO_4$-based** electrolyte. Mass loading ~2 mg cm$^{-2}$. Composition 60:30:10 wt.%.

__Note:__ In $LiClO_4$, $R_s$ is nearly invariant (~7 Ω), whereas $R_{ct}$ remains low and relatively flat (~225–255 Ω) across both charging and discharging, with only a slight increase at deep discharge.

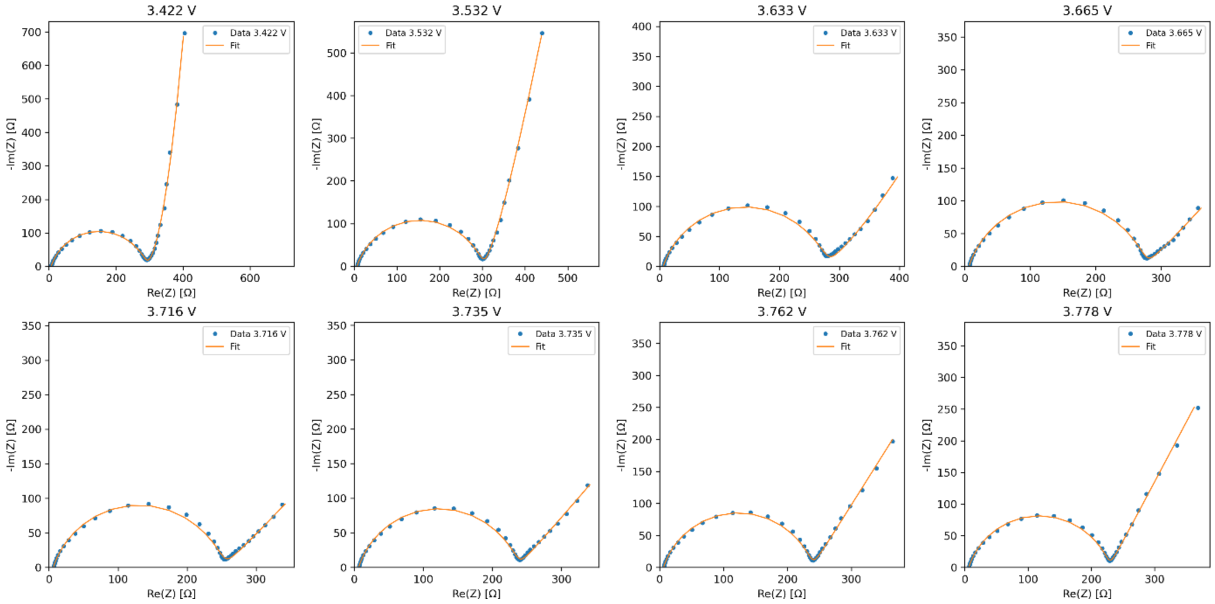


**Figure S22.** Nyquist plots at different potentials during the charging and discharging steps for **TEMPO-TP** COF buckypaper electrode in Li half-cell in **$BF_4$-based** electrolyte. Mass loading ~2 mg cm$^{-2}$. Composition 60:30:10 wt.%.

**<u>Note:</u>** For the $LiBF_4$-based electrolyte, $R_s$ is likewise nearly potential-independent at (~6.2–6.5 Ω), whereas $R_{ct}$ shows a moderate U-shaped dependence on state of charge: it decreases from ~280 Ω at the beginning of charge (3.52 V) to a minimum of ~210–230 Ω across the main TEMPO redox region (3.70–3.81 V), and then increases again during discharge to ~260–280 Ω at the deeply discharged state (3.42 V).

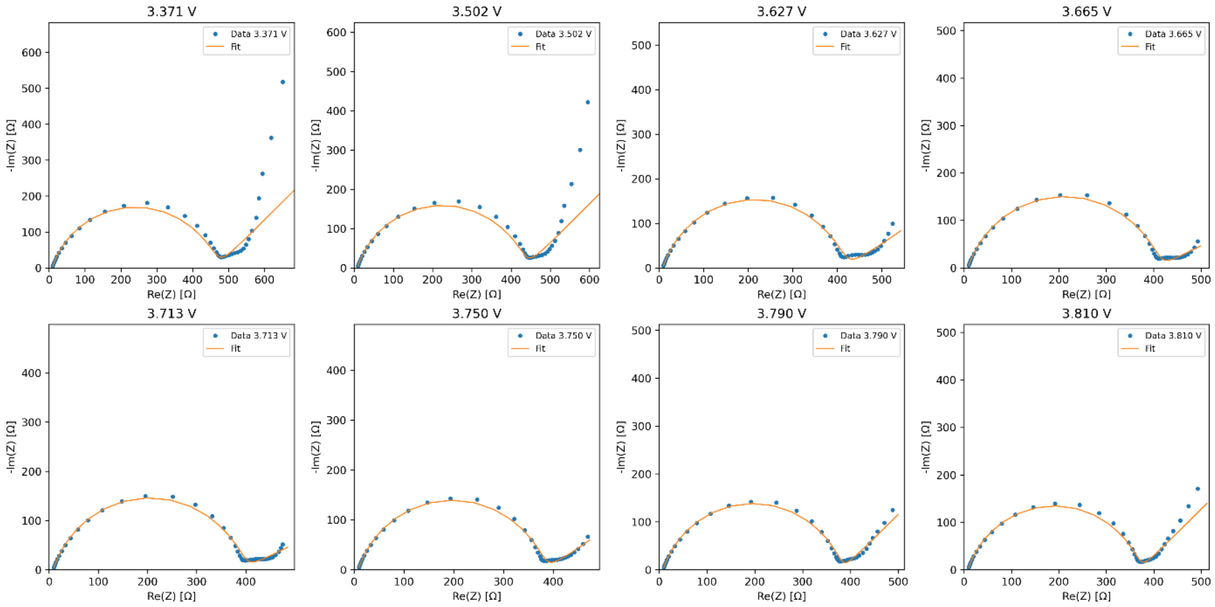


**Figure S23.** Nyquist plots at different potentials during the charging and discharging steps for **TEMPO-TP** COF buckypaper electrode in Li half-cell in **<u>DFOB-based</u>** electrolyte. Mass loading ~2 mg $cm^{-2}$. Composition 60:30:10 wt.%.

**<u>Note:</u>** For the LiDFOB-based electrolyte, $R_s$ remains low and nearly invariant at approximately ~6.8–7.3 Ω, whereas $R_{ct}$ exhibits a moderate state-of-charge dependence, decreasing from about ~420 Ω at the beginning of charge (3.50 V) to ~350 Ω across the main TEMPO oxidation region (3.71–3.81 V), remaining similarly low during the early discharge states (3.79–3.75 V), and then increasing again toward deep discharge to ~450 Ω at 3.37 V.

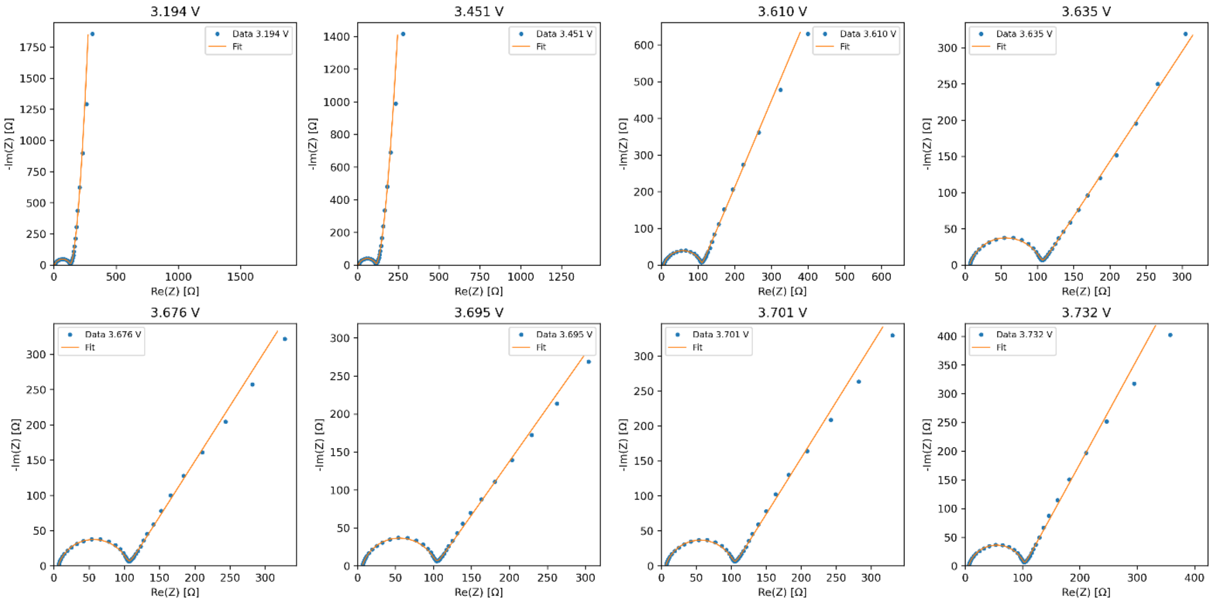


**Figure S24.** Nyquist plots at different potentials during the charging and discharging steps for **TEMPO-TP** COF buckypaper electrode in Li half-cell in **TFSI-based** electrolyte. Mass loading ~2 mg cm$^{-2}$. Composition 60:30:10 wt.%.

**Note:** For the LiTFSI-based electrolyte, $R_s$ remains essentially constant at approximately ~6.2–6.4 Ω, while $R_{ct}$ is the lowest and most weakly potential-dependent among the tested electrolytes, remaining in the range of roughly ~90–120 Ω over most of the charge/discharge process and increasing only slightly at deep discharge (3.19 V).

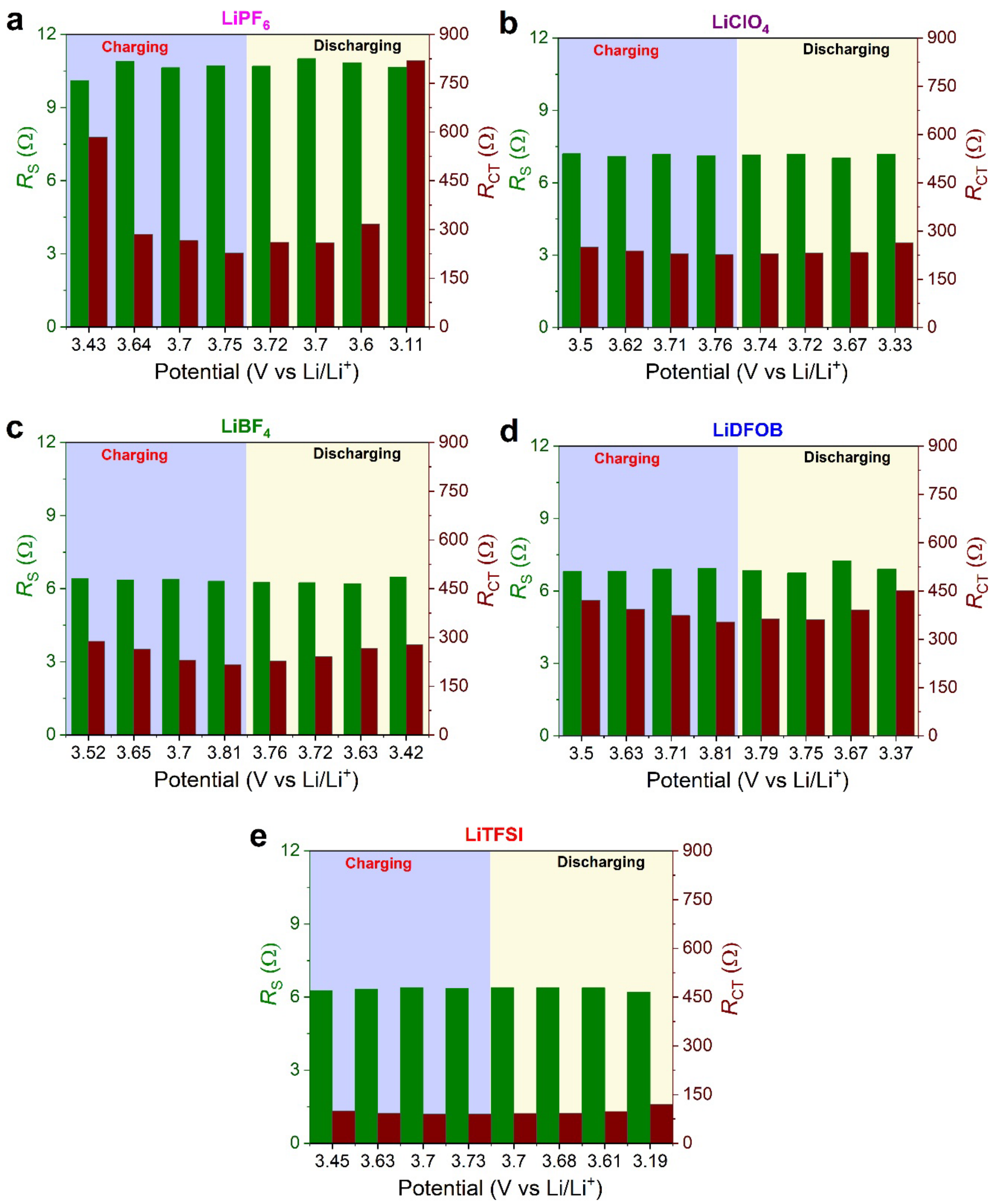


**Figure S25.** Nyquist Evolution of equivalent series resistance ($R_s$) and charge-transfer resistance ($R_{ct}$) for **TEMPO-TP** COF buckypaper electrode in Li half-cell at different potentials during the discharging and charging steps in a) $LiPF_6$, b) $LiClO_4$, c) $LiBF_4$, d) LiDFOB, and e) LiTFSI electrolytes.

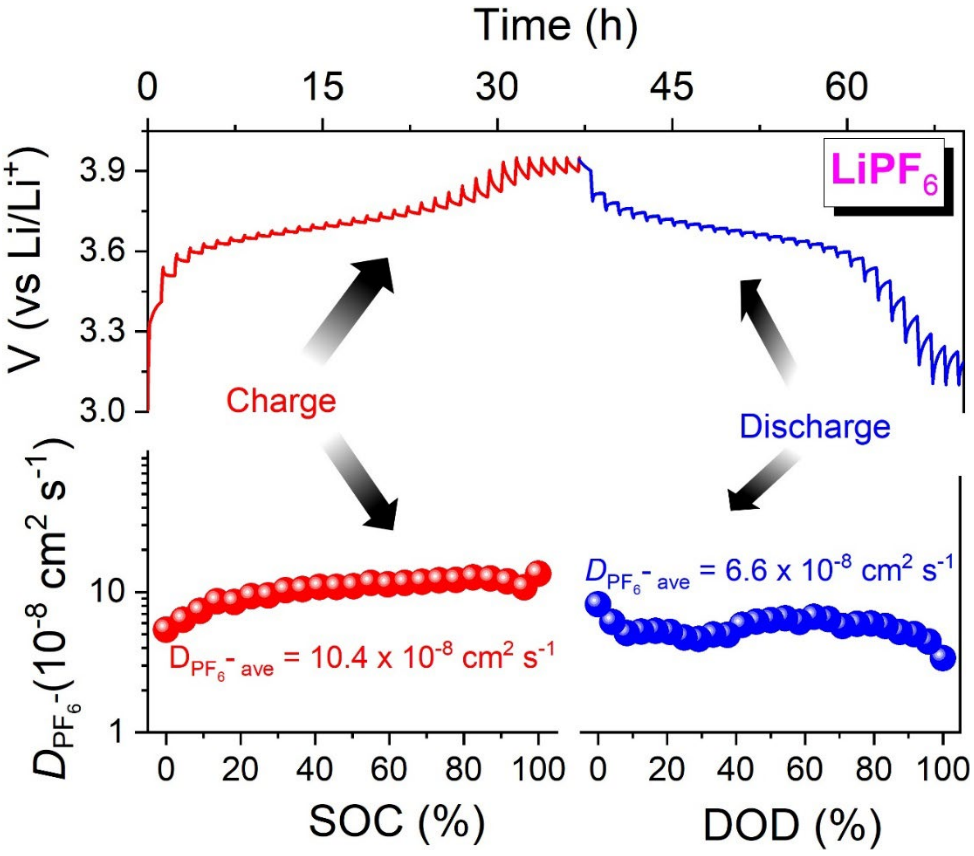


**Figure S26.** $D_{anion}$ ($D_{PF_6^-}$) diffusivity as a function of SoC/DoD (bottom range) calculated from GITT measurement (top range) for a **TEMPO-TP** COF buckypaper electrode in Li half-cell in $LiPF_6$ electrolyte. Mass loading ~2 mg cm$^{-2}$. Composition 60:30:10 wt.%.

**Note:** The extracted $D_{PF_6^-}$ values remain in the $10^{-8}$ cm$^2$ s$^{-1}$ range throughout both charging and discharging, with an average diffusivity of 10.4 × $10^{-8}$ cm$^2$ s$^{-1}$ during charge and 6.6 × $10^{-8}$ cm$^2$ s$^{-1}$ during discharge. During charging, $D_{PF_6^-}$ increases progressively with SoC and reaches its highest values in the main oxidation region, whereas during discharge it is systematically lower and shows a flatter dependence on DoD, followed by a decline near deep discharge.

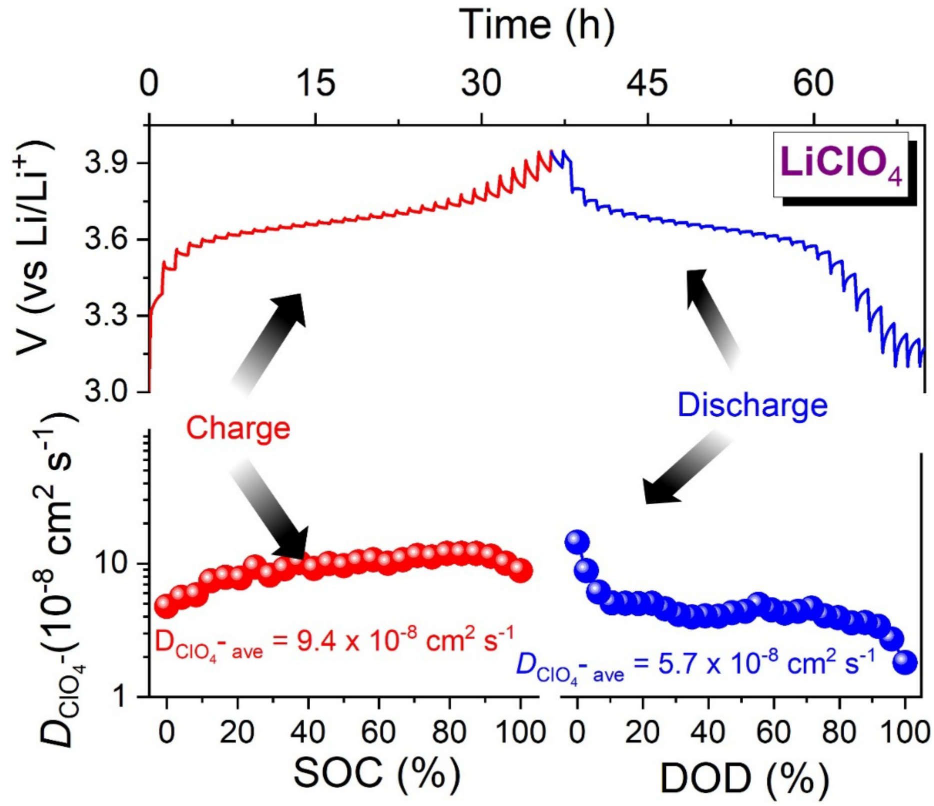


**Figure S27.** $D_{\mathrm{anion}}$ ( $D_{\mathrm{ClO_4^-}}$) diffusivity as a function of SoC/DoD (bottom range) calculated from GITT measurement (top range) for a **TEMPO-TP** COF buckypaper electrode in Li half-cell $LiClO_4$ electrolyte. Mass loading ~2 mg cm$^{-2}$. Composition 60:30:10 wt.%.

<u>**Note:**</u> For the $LiClO_4$-based electrolyte, the apparent anion diffusivity remains on the order of $10^{-8}$ cm$^2$ s$^{-1}$ throughout cycling, with an average value of $9.4 \times 10^{-8}$ cm$^2$ s$^{-1}$ during charge and $5.7 \times 10^{-8}$ cm$^2$ s$^{-1}$ during discharge; in general, the diffusivity increases slightly with SoC during charging, whereas it is lower and gradually decreases with DoD during discharging.

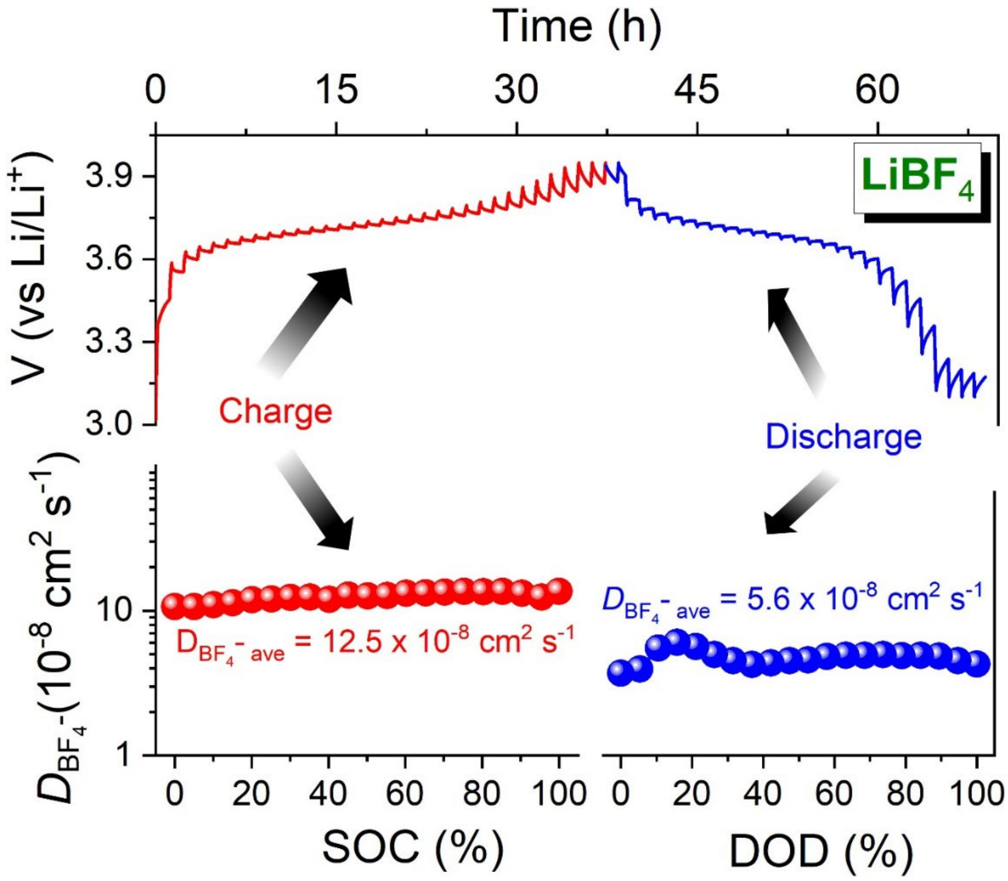


**Figure S28.** $D_{\text{anion}}$ ($D_{\mathrm{BF_4^-}}$) diffusivity as a function of SoC/DoD (bottom range) calculated from GITT measurement (top range) for a **TEMPO-TP** COF buckypaper electrode in Li half-cell $LiBF_4$ electrolyte. Mass loading ~2 mg $cm^{-2}$. Composition 60:30:10 wt.%.

__Note:__ For the $LiBF_4$-based electrolyte, the apparent anion diffusivity remains in the $10^{-8}$ $cm^2$ $s^{-1}$ range, with an average value of $12.5 \times 10^{-8}$ $cm^2$ $s^{-1}$ during charge and $5.6 \times 10^{-8}$ $cm^2$ $s^{-1}$ during discharge; overall, the diffusivity is relatively high and nearly constant during charging, whereas it is lower and shows only a weak variation with DoD during discharging.

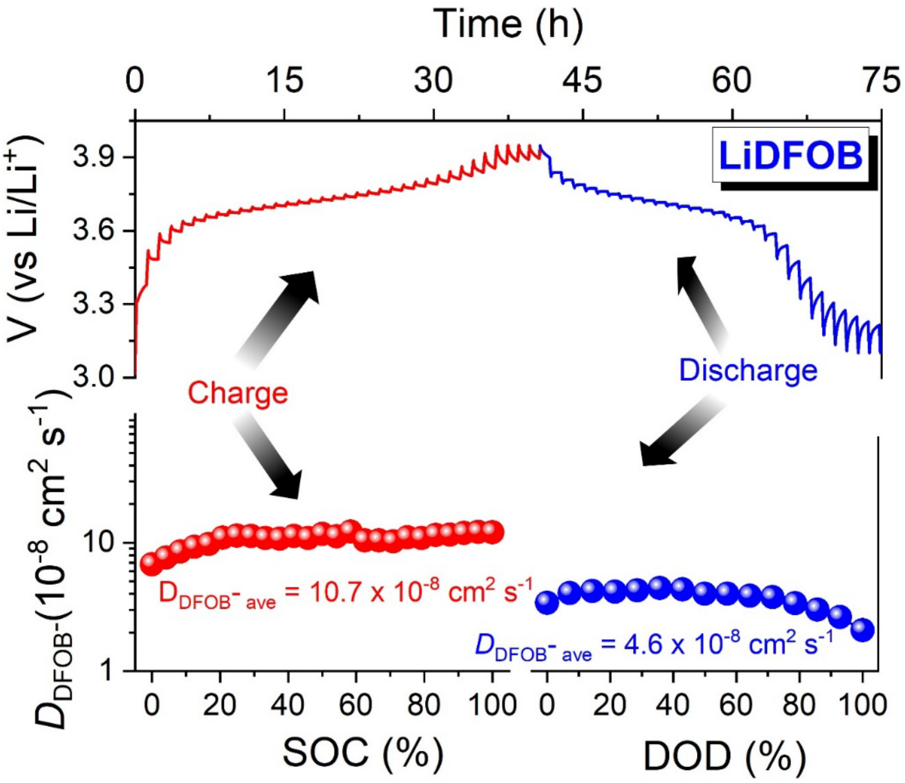


**Figure S29.** $D_{\text{anion}}$ ($D_{\text{DFOB}^-}$) diffusivity as a function of SoC/DoD (bottom range) calculated from GITT measurement (top range) for a **TEMPO-TP** COF buckypaper electrode in Li half-cell $LiBF_4$ electrolyte. Mass loading ~2 mg cm$^{-2}$. Composition 60:30:10 wt.%.

__**Note:**__ For the LiDFOB-based electrolyte, the apparent anion diffusivity remains in the $10^{-8}$ cm$^2$ s$^{-1}$ range, with an average value of $10.7 \times 10^{-8}$ cm$^2$ s$^{-1}$ during charge and $4.6 \times 10^{-8}$ cm$^2$ s$^{-1}$ during discharge; overall, the diffusivity increases initially and then remains relatively stable during charging, whereas it is lower during discharging and gradually decreases toward deep discharge.

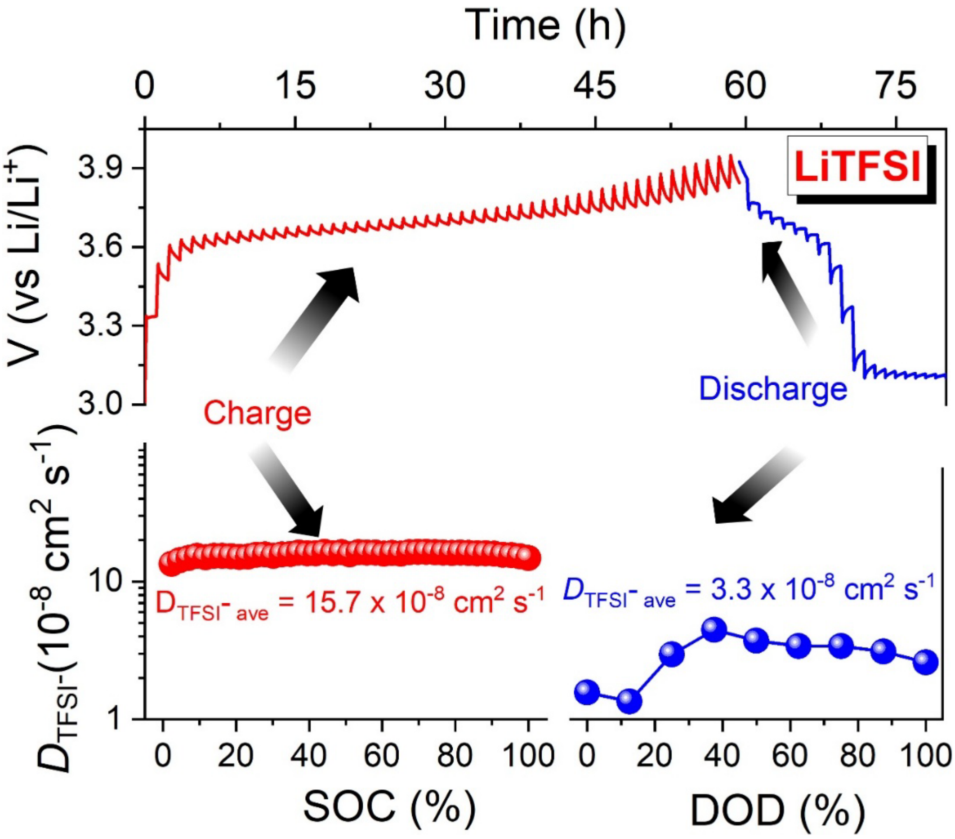


**Figure S30.** $D_{\text{anion}}$ ($D_{\text{TFSI}^-}$) diffusivity as a function of SoC/DoD (bottom range) calculated from GITT measurement (top range) for a **TEMPO-TP** COF buckypaper electrode in Li half-cell $LiBF_4$ electrolyte. Mass loading ~2 mg cm$^{-2}$. Composition 60:30:10 wt.%.

__Note:__ For the LiTFSI-based electrolyte, the apparent anion diffusivity remains in the $10^{-8}$ cm$^2$ s$^{-1}$ range, with an average value of $15.7 \times 10^{-8}$ cm$^2$ s$^{-1}$ during charge and $3.3 \times 10^{-8}$ cm$^2$ s$^{-1}$ during discharge; overall, the diffusivity is relatively high and nearly constant during charging, whereas it is much lower during discharging, showing an initial increase followed by a gradual decline at higher DoD.

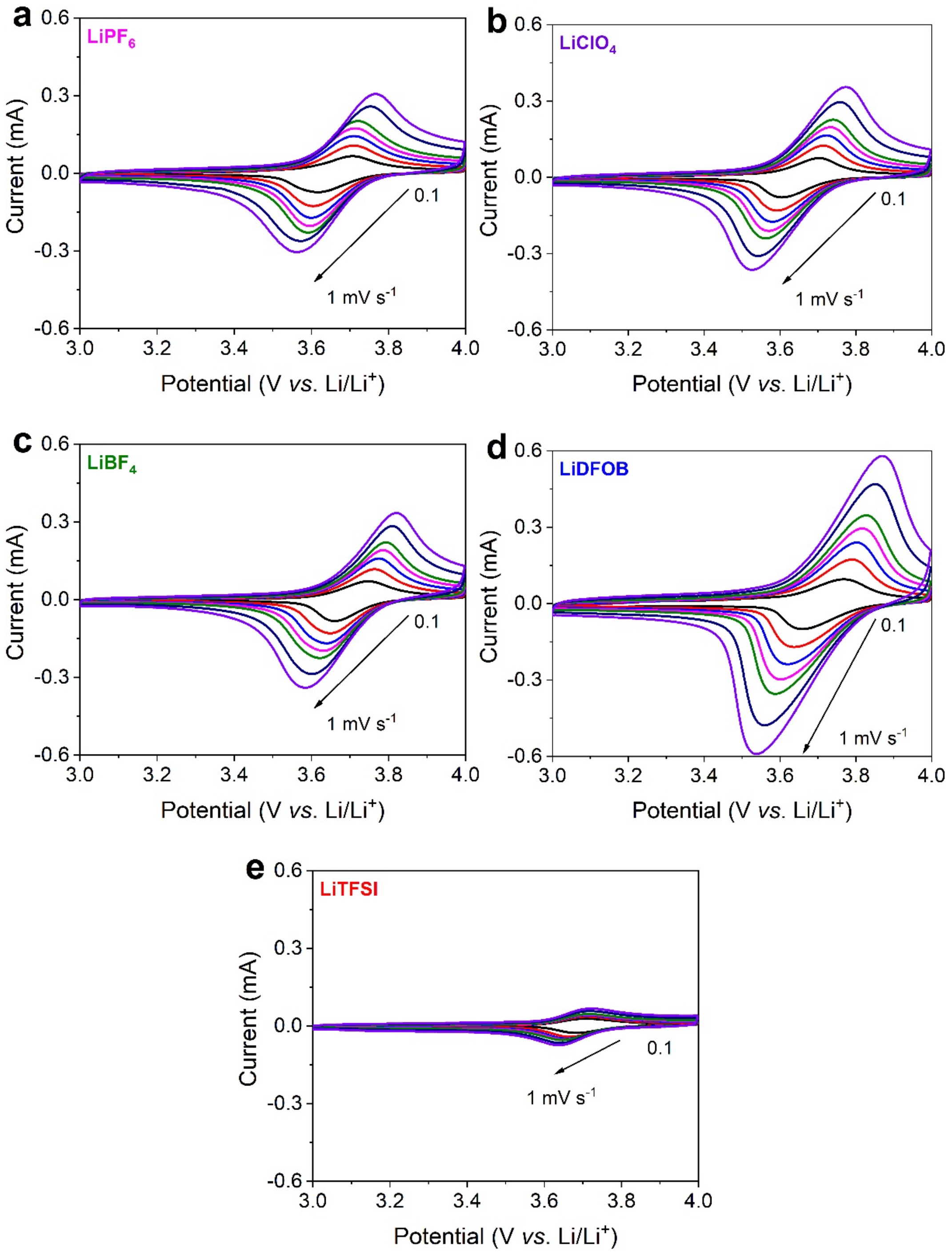


**Figure S31.** CVs at different scan rates for **TEMPO-TP** COF buckypaper electrode in Li half-cell in a) $LiPF_6$, b) $LiClO_4$, c) $LiBF_4$, d) LiDFOB, and e) LiTFSI electrolytes. Mass loading ~2 mg $cm^{-2}$. Composition 60:30:10 wt.%.

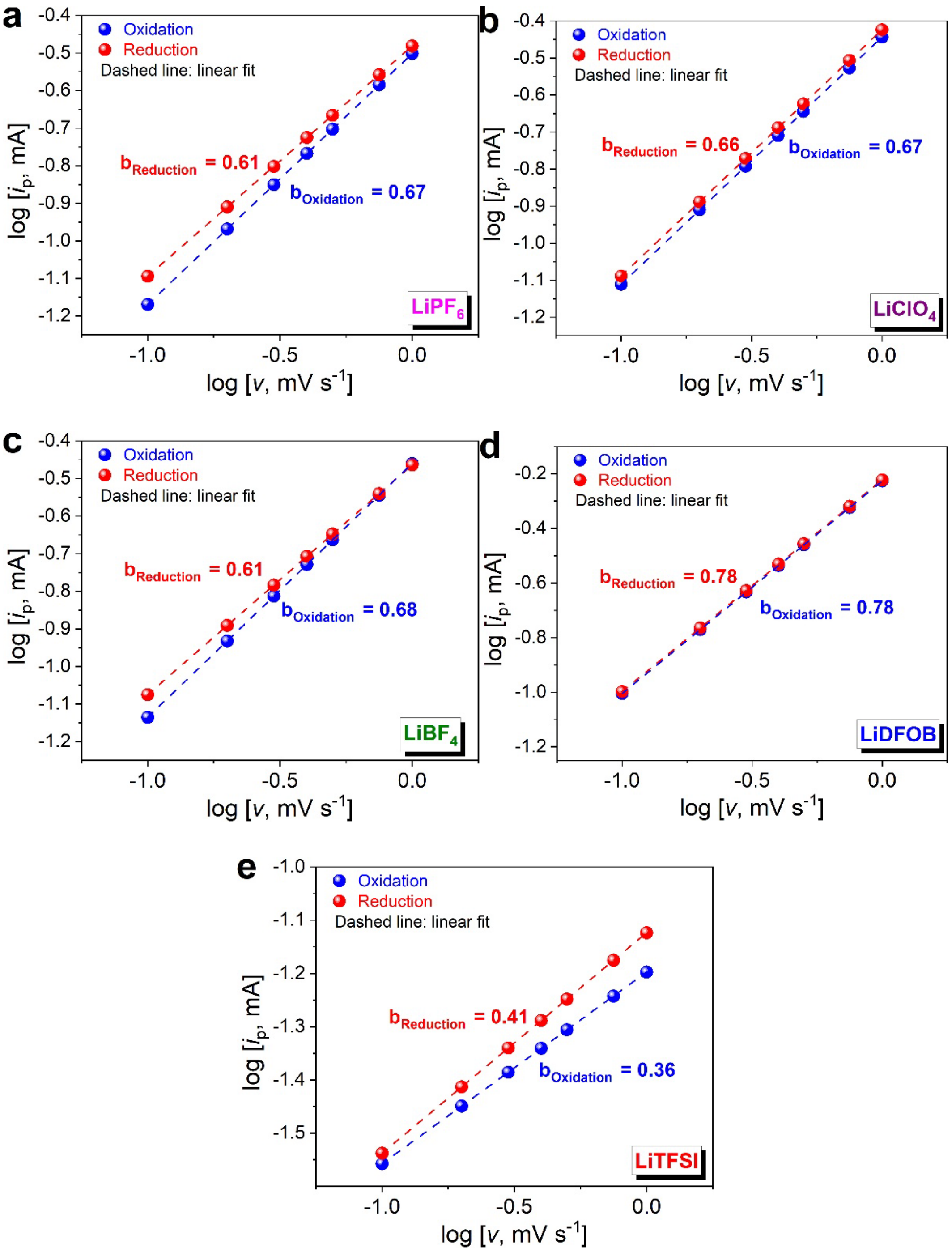


**Figure S32.** Peak current vs scan rate in logarithmic scale to obtain b-values according to $i_p = av^b$ for **TEMPO-TP** COF buckypaper electrode in Li half-cell at different scan rates in a) $LiPF_6$, b) $LiClO_4$, c) $LiBF_4$, d) LiDFOB, and e) LiTFSI electrolytes. Mass loading ~2 mg cm$^{-2}$. Composition 60:30:10 wt.%.

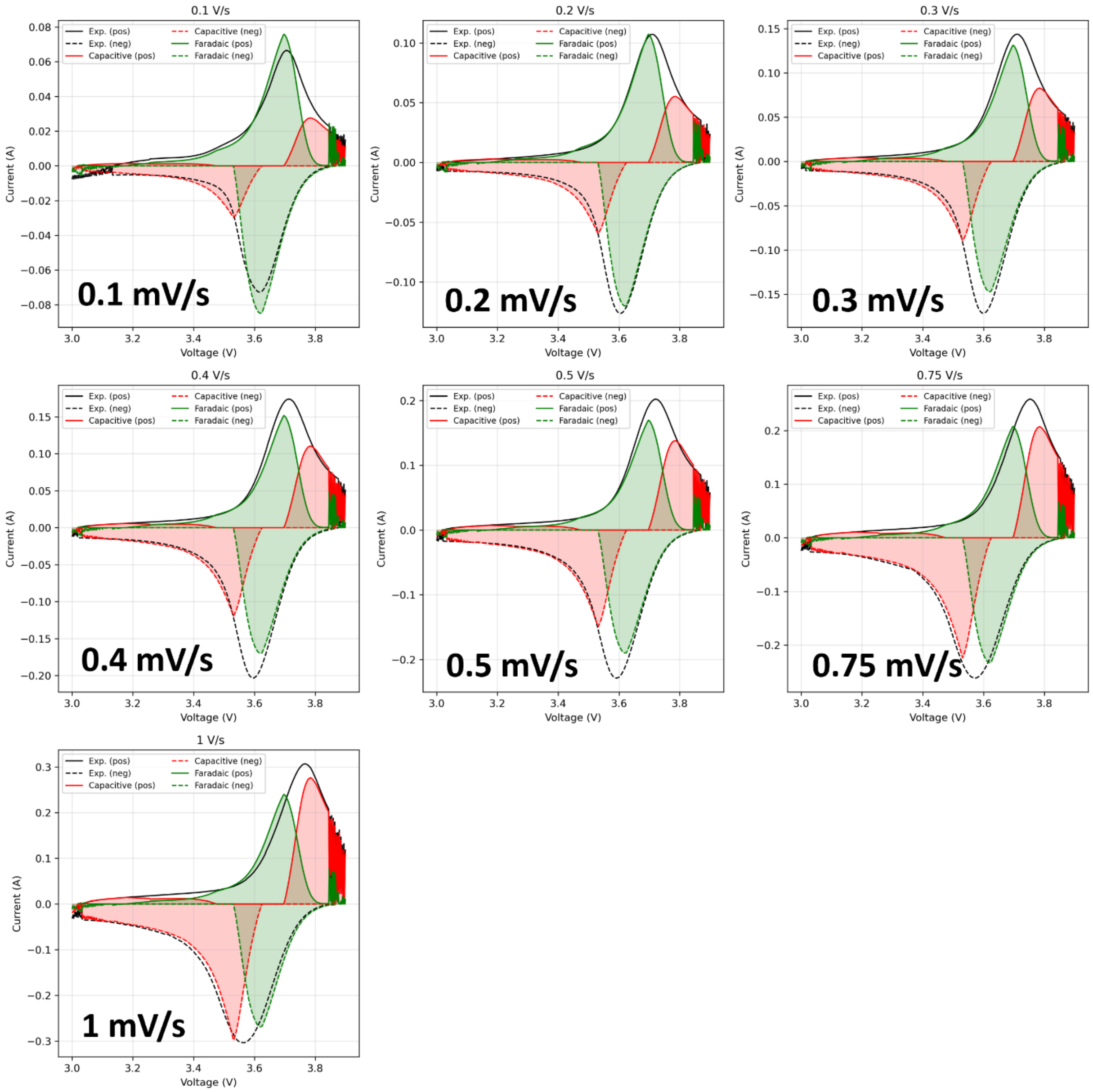


**Figure S33.** Surface-, diffusion-controlled and total current contributions in CV at different scan rates for **TEMPO-TP** COF buckypaper electrode in $LiPF_6$ based half-cell. Mass loading ~2 mg $cm^{-2}$. Composition 60:30:10 wt.%.

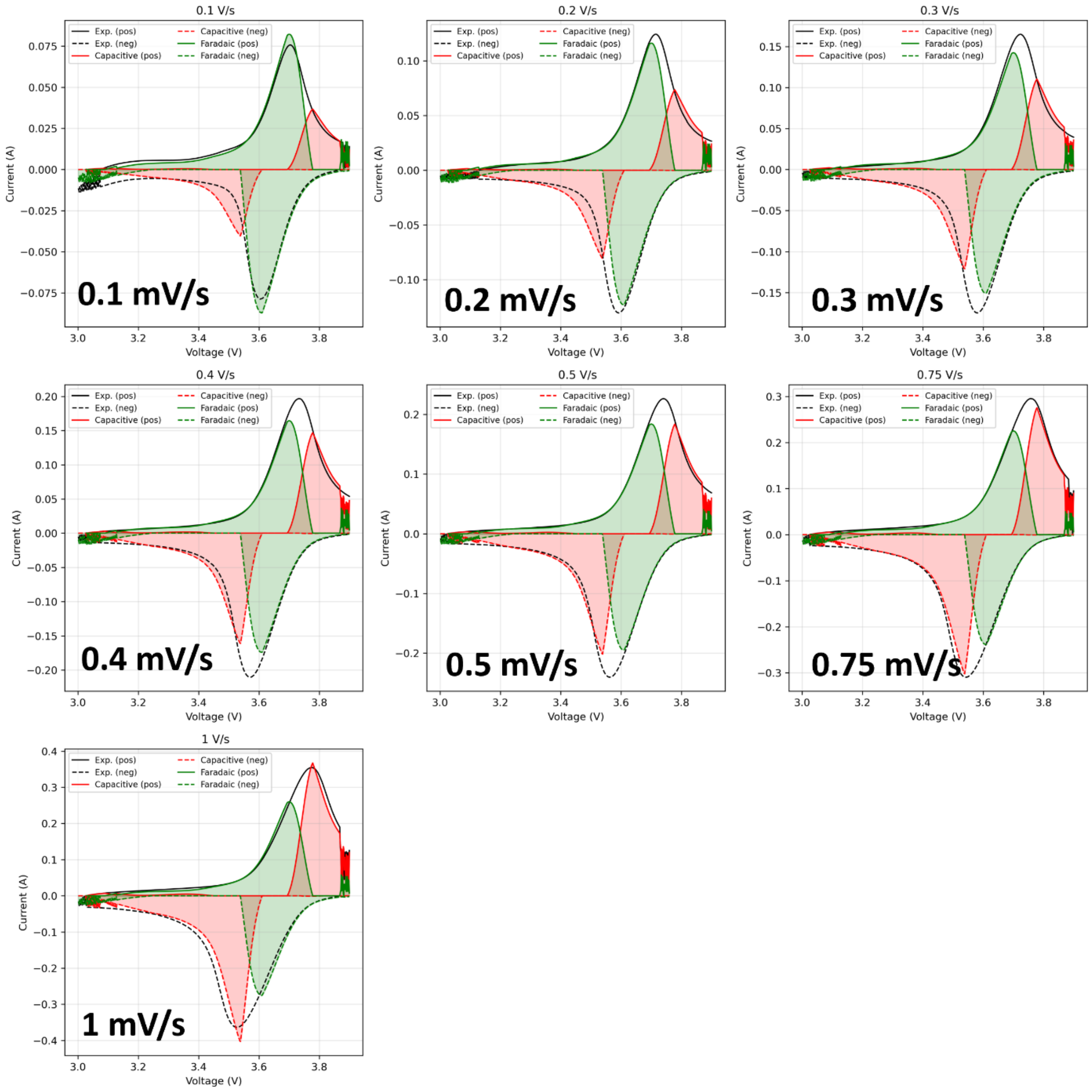


**Figure S34.** Surface-, diffusion-controlled and total current contributions in CV at different scan rates for **TEMPO-TP** COF buckypaper electrode in $LiClO_4$ based half-cell. Mass loading ~2 mg cm$^{-2}$. Composition 60:30:10 wt.%.

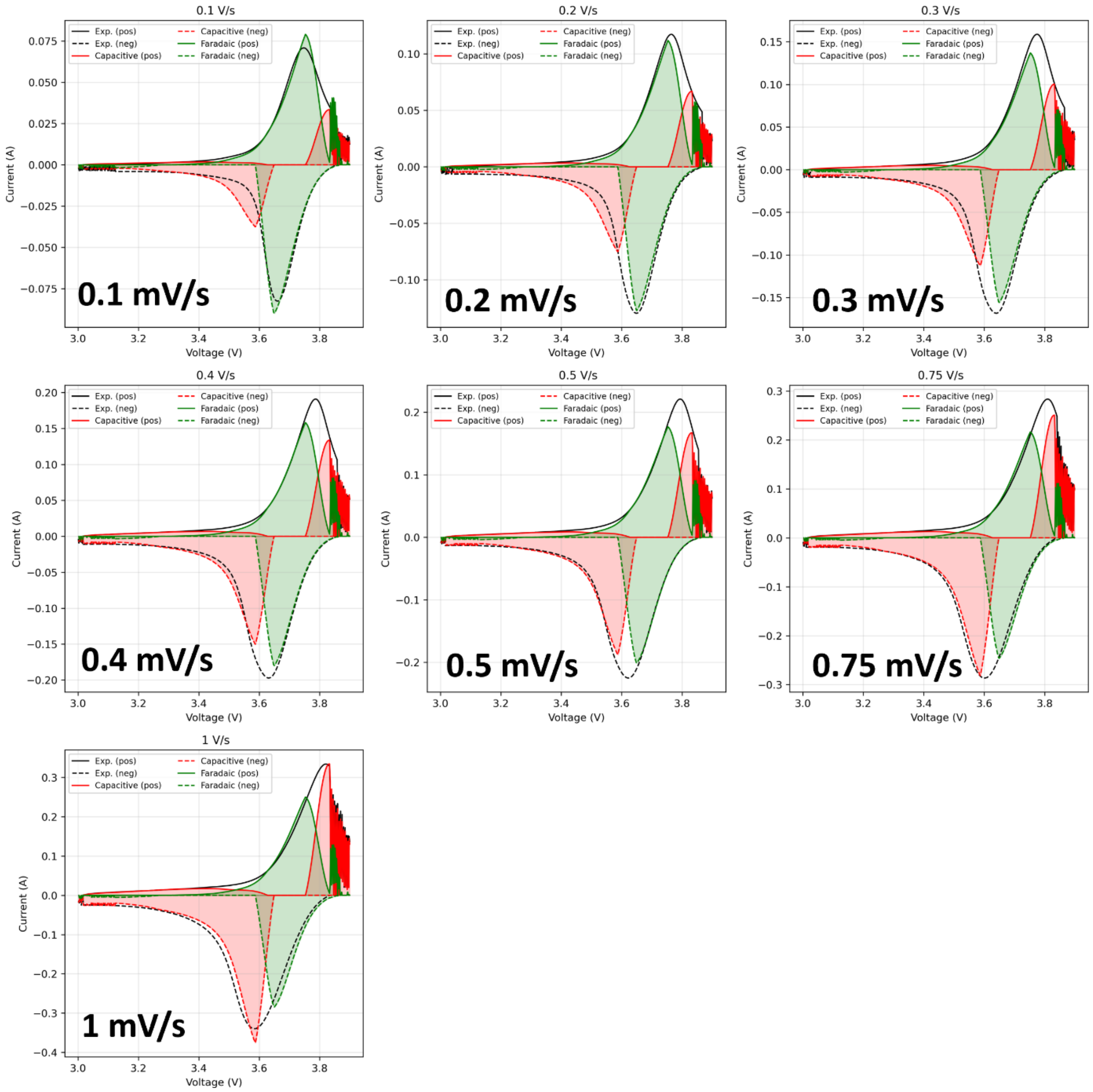


**Figure S35.** Surface-, diffusion-controlled and total current contributions in CV at different scan rates for **TEMPO-TP** COF buckypaper electrode in $LiBF_4$ based half-cell. Mass loading ~2 mg cm$^{-2}$. Composition 60:30:10 wt.%.

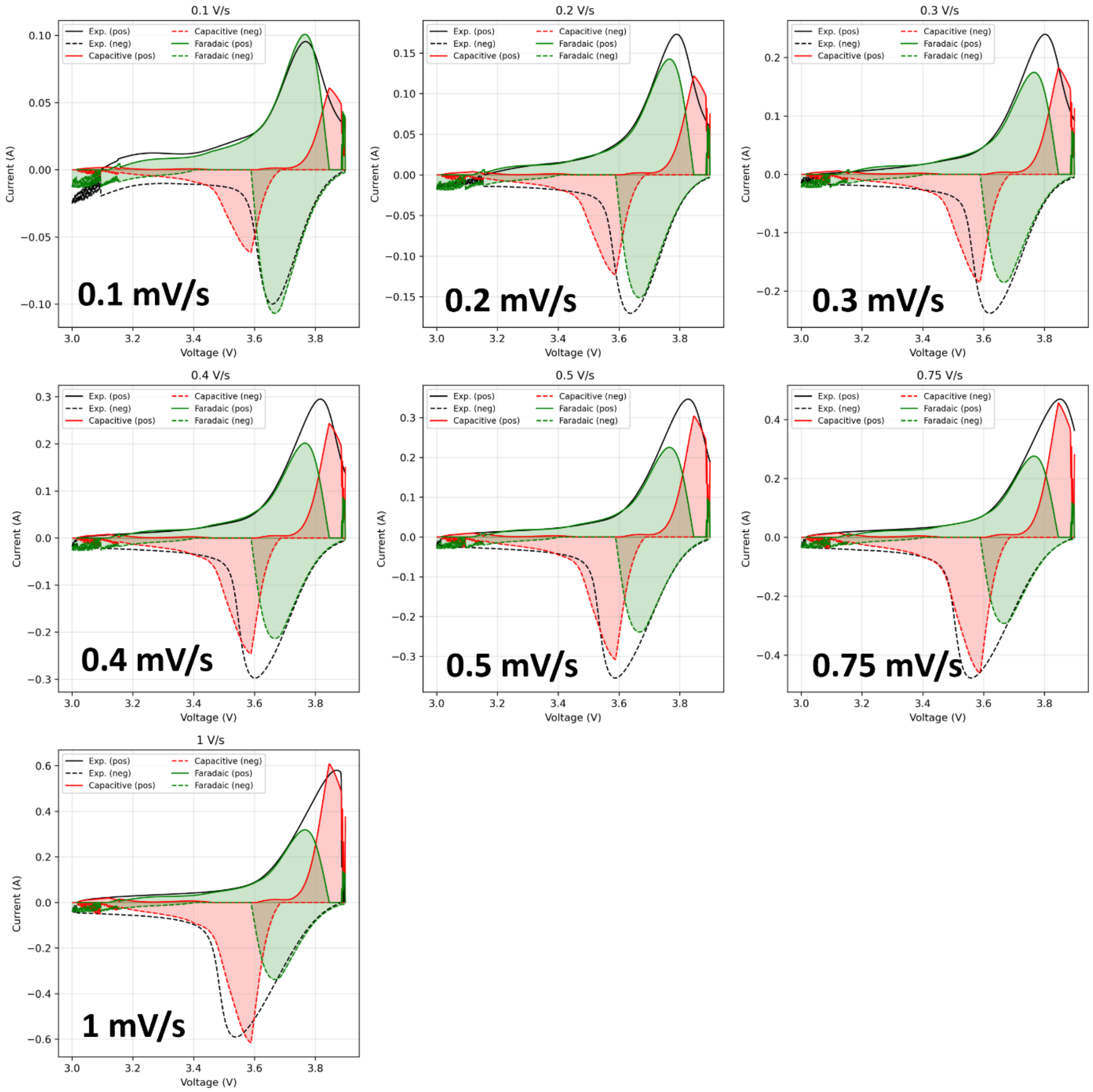


**Figure S36.** Surface-, diffusion-controlled and total current contributions in CV at different scan rates for **TEMPO-TP** COF buckypaper electrode in LiDFOB based half-cell. Mass loading ~2 mg $cm^{-2}$. Composition 60:30:10 wt.%.

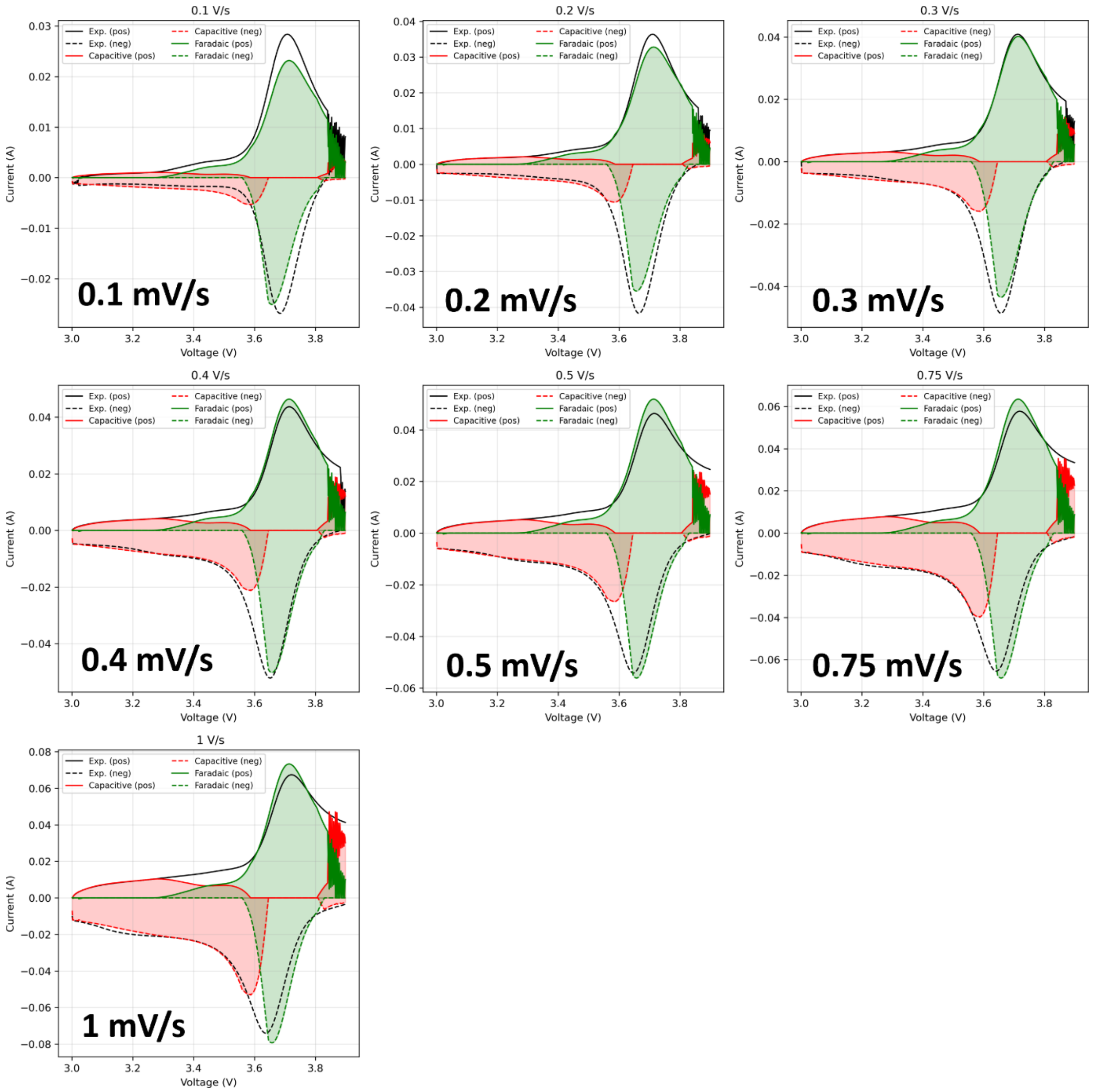


**Figure S37.** Surface-, diffusion-controlled and total current contributions in CV at different scan rates for **TEMPO-TP** COF buckypaper electrode in LiTFSI based half-cell. Mass loading ~2 mg cm$^{-2}$. Composition 60:30:10 wt.%.

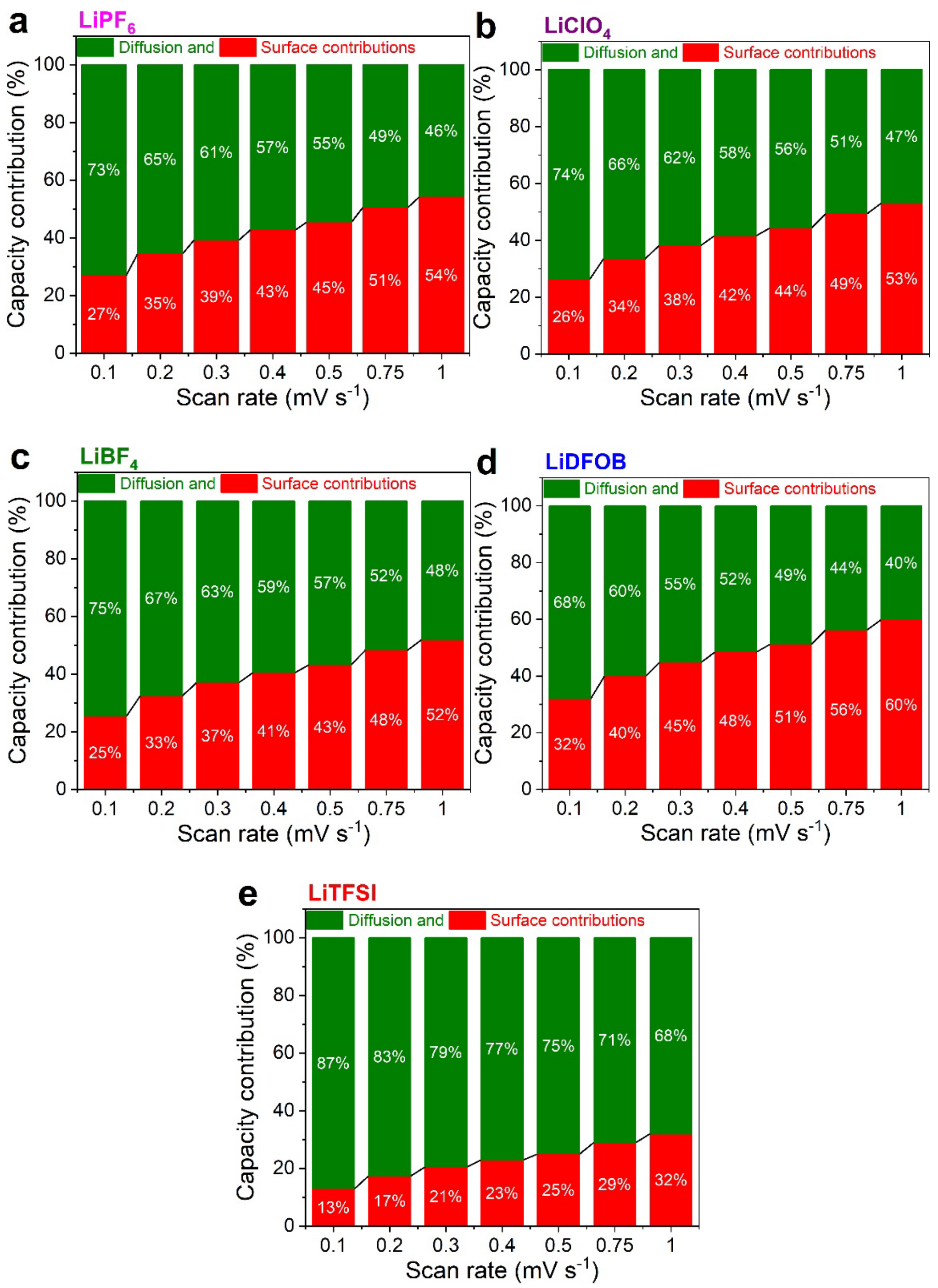


**Figure S38.** Surface- and diffusion-contributions calculated applying the Dunn's method for **TEMPO-TP** COF buckypaper electrode in Li half-cell at different scan rates in a) $LiPF_6$, b) $LiClO_4$, c) $LiBF_4$, d) LiDFOB, and e) LiTFSI electrolytes. Mass loading ~2 mg cm$^{-2}$. Composition 60:30:10 wt.%.

**Table S1.** Comparison of capacity at 1st cycle and capacity at 200 cycle, capacity retention (%) and % decay per cycle.

| Material | Anion | Capacity (1st cycle) | Capacity (200 cycle) | Capacity retention % | % decay | Average *CE* |
|---|---|---|---|---|---|---|
| TEMPO-TP COF | $PF_6$ | 30.9 | 14.9 | 58.0 | 51.7 | 99.6 |
| | $ClO_4^-$ | 36.7 | 23.6 | 64.6 | 35.6 | 98.4 |
| | $BF_4$ | 32.8 | 18.9 | 62.6 | 42.3 | 99.1 |
| | DFOB | 36.4 | 23.0 | 68.7 | 36.8 | 99.4 |
| | TFSI | 22.4 | 10.6 | 37.7 | 52.6 | 98.1 |
| TEMPO-TB COF | $PF_6$ | 39.7 | 23.5 | 60.5 | 40.8 | 99.6 |
| | $ClO_4^-$ | 41.1 | 24.9 | 60.6 | 39.4 | 98.7 |
| | $BF_4$ | 37.6 | 21.5 | 59.3 | 42.8 | 99.6 |
| | DFOB | 40.9 | 29.0 | 72.1 | 29.0 | 99.1 |
| | TFSI | 34.0 | 13.5 | 40.0 | 60.2 | 94.5 |

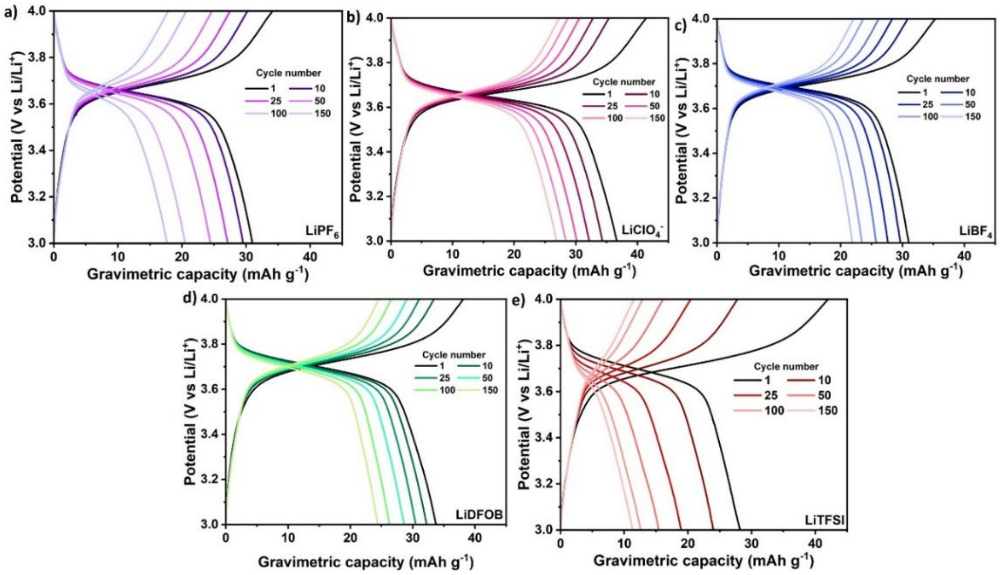


**Figure S39.** Representative GCD profiles of the **TEMPO-TP** COF electrode at different cycle number in a) $LiPF_6$, b) $LiClO_4$, c) $LiBF_4$, d) LiDFOB and e) LiTFSI electrolytes upon cycling at 1C. Mass loading ~2 mg $cm^{-2}$. Composition 60:30:10 wt.%.

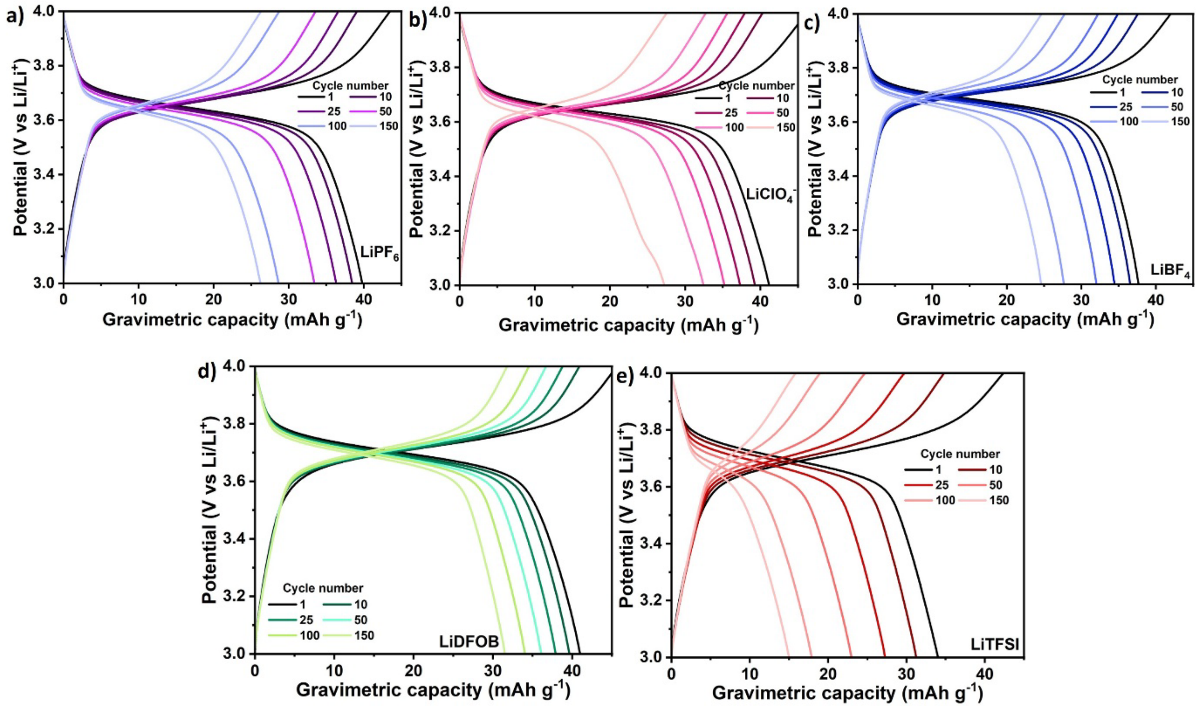


**Figure S40.** Representative GCD profiles of the **TEMPO-TB** COF electrode at different cycle number in a) $LiPF_6$, b) $LiClO_4$, c) $LiBF_4$, d) LiDFOB and e) LiTFSI electrolytes upon cycling at 1C. Mass loading ~2 mg cm$^{-2}$. Composition 60:30:10 wt.%.

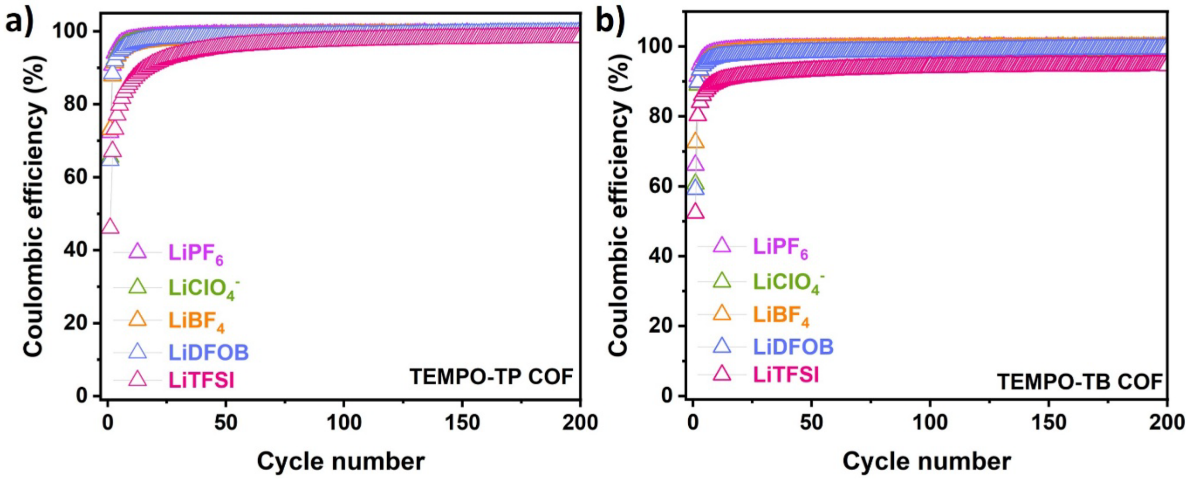


**Figure S41.** CE vs cycle number of a) **TEMPO-TP** and b) **TEMPO-TB** COFs. Mass loading ~2 mg cm$^{-2}$. Composition 60:30:10 wt.%.

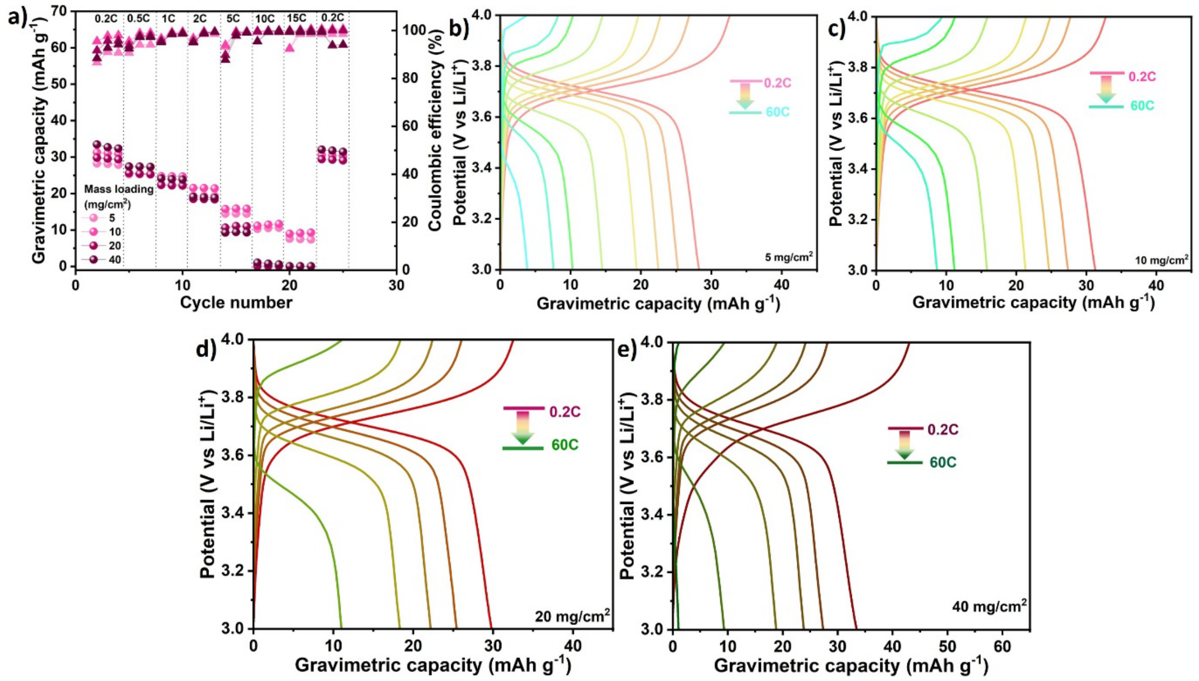

**Figure S42.** a) Capacity utilization vs C-rates; representative GCPL profile of the **TEMPO-TB** COF electrode using different mass loading: b) 5 mg/cm$^2$, c) 10 mg/cm$^2$, d) 20 mg/cm$^2$ and e) 40 mg/cm$^2$. Electrode composition 80:15:5 wt.%.

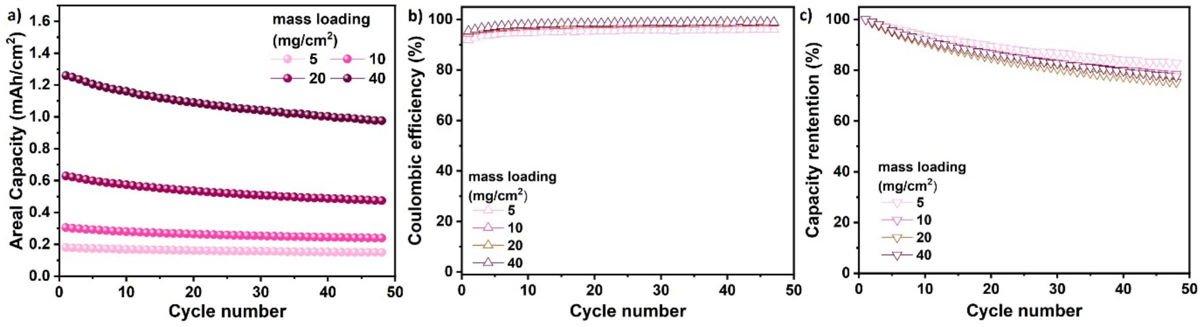

**Figure S43.** Cycle stability evaluation of **TEMPO-TP COF** buckypaper electrodes with different mass loadings (5, 10, 20 and 40 mg/cm$^2$) in Li half-cells conducted at 1C. Electrode composition 80:15:5 wt.%. a) areal capacity, b) Coulombic efficiency and c) capacity retention.

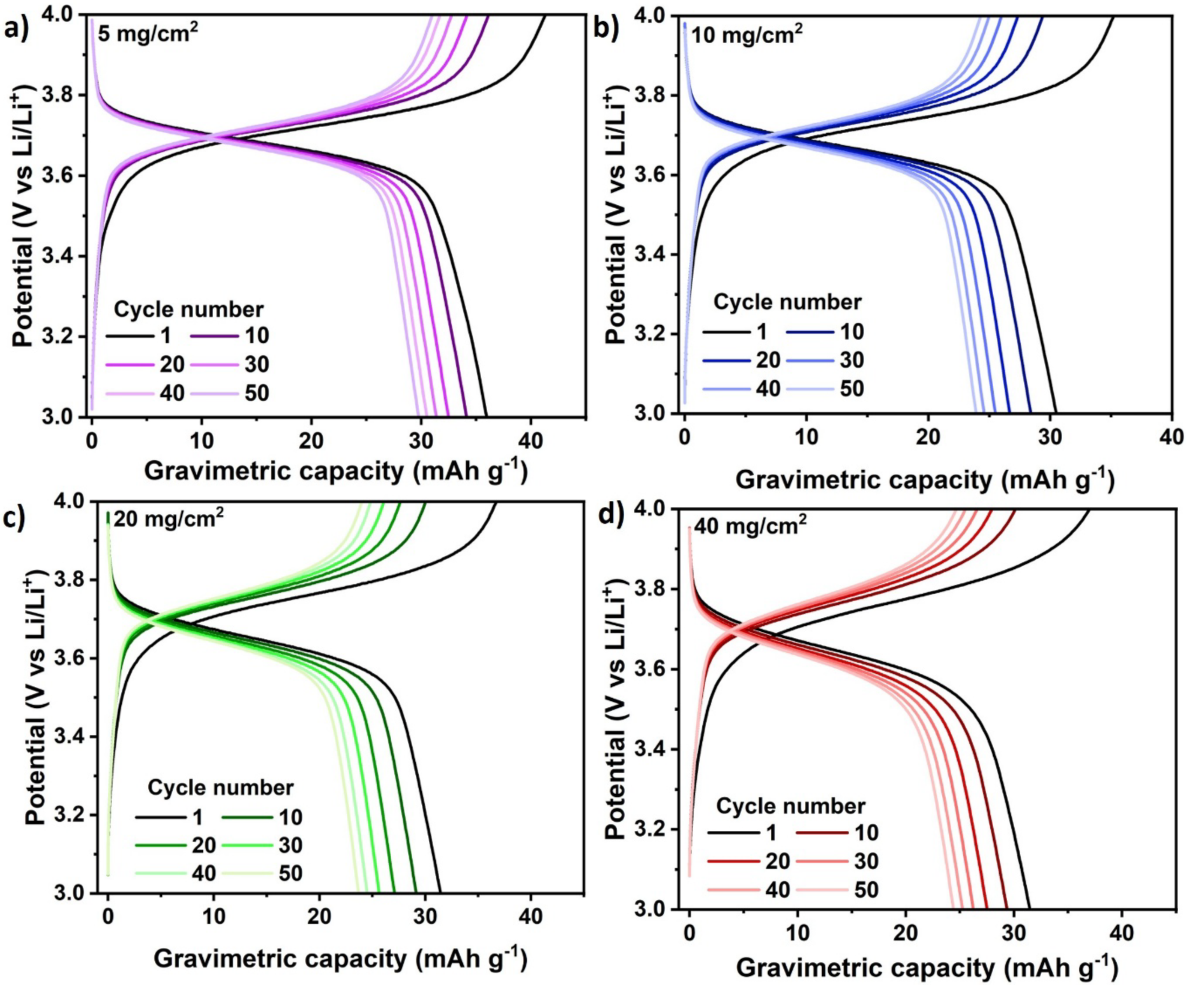


**Figure S44.** Representative GCD profiles of the **TEMPO-TP COF** electrode at different cycle number in LiDFOB electrolyte upon cycling at 1C. Electrode composition 80:15:5 wt.%.

**Table S2.** A list of state-of-the-art Li || TEMPO/PTMA based Li half-cells, mainly reporting gravimetric and areal capacities.

| S.No | TEMPO/PTMA cathode | Mass loading ($mg/cm^2$) | Active material content (wt.%) | C rate | Gravimetric capacity (mAh $g^{-1}$) | Areal capacity (mAh $cm^{-2}$) | Ref. |
|---|---|---|---|---|---|---|---|
| **1.** | PTVE | 2.2 | 80 | 1 | 104 | 0.23 | 3 |
| **2.** | PTMA | 2 | 40 | 30 | 75 | 0.1 | 4 |
| **3.** | TEMPO based di radical | 0.95 | 40 | 1 | 68 | 0.06 | 5 |
| **4.** | PTMA hybrid-HYB 11 | 3.6 | 84 | 0.5 | 47 | 0.39 | 6 |
| **5.** | PTMA:poly[Ni(MeSalen)] | 0.2 | 25 | 1 | 83 | 0.016 | 7 |
| **6.** | PTMA-MW2.5 | 4.28 | 85.6 | 0.2 | 93.2 | 0.387 | 8 |
| **7.** | PTMA-MW15 | 9.01 | 75.1 | 0.2 | 99.8 | 0.410 | 8 |
| **8.** | TPTPA | - | - | - | 105.7 | - | 9 |
| **9.** | **TEMPO-TP** | **2** | **60** | **0.2** | **38** | **0.076** | **this work** |
| **10.** | **TEMPO-TP** | **40** | **80** | **0.2** | **33.46** | **1.3** | **this work** |

**Note:** This comparison focuses on the peak values of gravimetric capacities of the TEMPO/PTMA-based cathodes to demonstrate the competitive standing of our material within the current literature landscape.

## 7. References


[1] V. Augustyn, J. Come, M. A. Lowe, J. W. Kim, P.-L.s Taberna, S. H. Tolbert, H. D. Abruña, P. Simon, B. Dunn. *Nat. Mater.*, **2013**, 12, 518–522.

[2] T. Brezesinski, J. Wang, S. H. Tolbert, B. Dunn. *Nat. Mater. 2010 92*, **2010**, 9, 146–151.

[3] M. Suguro, S. Iwasa, K. Nakahara. *Macromol. Rapid Commun.* **2008**, *29*, 1635–1639.

[4] J.-K. Kim, J.-H. Ahn, G. Cheruvally, G. S. Chauhan, J.-W. Choi, D.-S. Kim, H.-J. Ahn, S. H. Lee, C. E. Song. *Met. Mater. Int.*, **2009**, *15*, 77-82.

[5] X. Li, G. Cheruvally, J.-K. Kim, J.-W. Choi, J.-H. Ahn, K.-W. Kim, H.-J. Ahn. *RSC Advances* **2012**, *2*, 10394–10399.

[6] G. Dolphijn, S. Isikli, F. Gauthy, A. Vlad, J.-F. Gohy. *Electrochim. Acta* **2017**, *255*, 442–448.

[7] A. A. Vereshchagin, P. S. Vlasov, A. S. Konev, P. Yang, G. A. Grechishnikova, O. V. Levin. *Electrochim. Acta* **2019**, *295*, 1075-1084.

[8] A. Innocenti, I. A. Moisés, O. Lužanin, J. Bitenc, J.-F. Gohy, S. Passerini. *ACS Appl. Mater. Interfaces* **2024**, *16*, 48757−48770.

[9] C. Zhang, X. Yang, W. Ren, Y. Wang, F. Su, J.-X. Jiang. *J. Power Sources* **2016**, *317*, 49-56.
